# Myosin IIA motor regulates attaching-effacing bacteria interactions with intestinal epithelium

**DOI:** 10.1080/19490976.2026.2638002

**Published:** 2026-02-28

**Authors:** Nayden G. Naydenov, Atif Zafar, Susana Lechuga, Ajay Zalavadia, Armando Marino-Melendez, John A. Hammer, Velia M. Fowler, Christine McDonald, Kenneth G. Campellone, Andrei I. Ivanov

**Affiliations:** a Department of Inflammation and Immunity, Cleveland Clinic Research, Cleveland Clinic, Cleveland, OH, USA; b Department of Molecular Medicine, Cleveland Clinic Lerner College of Medicine at Case Western Reserve University, Case Western Reserve University, Cleveland, OH, USA; c Imaging Core, Cleveland Clinic Research, Cleveland Clinic, Cleveland, OH, USA; d Cell and Developmental Biology Center, National Heart, Lung and Blood Institute, National Institutes of Health, Bethesda, MD, USA; e Department of Biological Sciences, University of Delaware, Newark, DE, USA; f Department of Molecular & Cell Biology and Institute for Systems Genomics, University of Connecticut, Storrs, CT, USA

**Keywords:** Myosin II, actin cytoskeleton, attaching/effacing bacteria, intestinal epithelium

## Abstract

Attaching effacing (A/E) bacteria, such as enteropathogenic *Escherichia coli* (EPEC) and *Citrobacter rodentium* colonize intestinal epithelial cells (IECs) by inducing remodeling of the epithelial cytoskeleton and the formation of prominent actin pedestals at bacterial attachment sites. While non-muscle myosin II (NM II) is a key regulator of the actin cytoskeleton, whether it regulates IEC colonization by A/E pathogens is not known. To address this question, we targeted NM IIA and NM IIC, the NM II paralogs expressed in IECs. Our *in vivo* studies utilized mouse models with either intestinal epithelial-specific deletion of NM IIA (NM IIA cKO mice), expression of a NM IIA motor domain mutant, or total deletion of NM IIC (NM IIC tKO mice). *In vitro* experiments utilized IECs (HT-29cF8 and Caco-2BBE) with CRISPR-Cas9-mediated deletion of NM IIA or NM IIC. In addition, NM II activity *in vitro* was modulated pharmacologically, using either the pan-myosin inhibitor, blebbistatin, or a specific NM IIC activator, 4-hydroxyacetophenone (4-HAP). NM IIA cKO and NM IIA mutant mice demonstrated higher *C. rodentium* colonization, along with more severe mucosal inflammation and colonic crypt hyperplasia as compared to their controls. By contrast, NM IIC tKO mice was indistinguishable from their control with regard to *C. rodentium* colonization. Blebbistatin treatment increased EPEC attachment to IECs monolayers, whereas 4-HAP did not affect bacterial attachment. Genetic knockout of NM IIA, but not NM IIC, increased EPEC adhesion to IEC monolayers. Importantly, the increase in EPEC attachment exhibited by NM IIA-deficient IECs required an intact bacterial Type 3 secretion system and functional Tir effector, indicating that NM IIA functions in actin pedestal assembly. In summary, we describe a novel role for NM IIA in limiting intestinal epithelial colonization by A/E pathogens via the inhibition of pathogen-induced remodeling of the actin cytoskeleton.

## Introduction

Remodeling of the actin cytoskeleton is a key mechanism utilized by different pathogenic microorganisms to colonize mammalian cells.^1^^,^^2^ Pathogens perturb the actin filament architecture and dynamics and trigger the assembly of new cytoskeletal structures to support their adhesion, invasion, and dissemination within host cells and tissues.^1^^,^^2^ A group of enteric pathogenic bacteria is known to induce dramatic remodeling of the mammalian actin cytoskeleton by attaching to the host cell surface and injecting different effector proteins that elicit a variety of intracellular signaling events. Such pathogens include Enteropathogenic *Escherichia coli* (EPEC) and Enterohemorrhagic *Escherichia coli* (EHEC), which are known to cause serious diarrheal diseases in children and immunocompromised adults.^3^^,^^4^ Similar features are also attributed to a mouse pathogen, *Citrobacter rodentium*, which is commonly used as an experimental model for EPEC and EHEC infection.^5^^,^^6^ Collectively, these bacteria are referred to as ‘attaching/effacing’ (A/E) bacteria owing to their ability to destroy microvilli and form attaching/effacing lesions at the apical surface of infected intestinal epithelial cells (IECs).^7-9^ The hallmark of A/E pathogen-induced remodeling of the host cytoskeleton is the assembly of actin-rich pedestals underneath the plasma membrane where attached bacteria reside.^1^^,^^2^^,^^10^ These pedestals represent the most vivid morphological alteration of host cells triggered by the attached pathogen, and they appear to be essential accelerators of bacterial infection.^11^

The ability of A/E pathogens to remodel the host cytoskeleton and build actin pedestals depends on crucial bacterial virulence factors, such as a type III secretion system (T3SS), the adhesin intimin, and its receptor Tir. The T3SS system represents a syringe-shaped multiprotein complex penetrating the host membrane that is used to inject many bacterial effector proteins into host cells.^12-14^ Bacterial Tir and other secreted effectors stimulate actin filament polymerization within the vicinity of the attached bacteria, leading to actin pedestal assembly.^12-14^ The mechanisms by which A/E pathogens subvert the host actin polymerization machinery have been extensively investigated over several decades.^10^^,^^15-18^ However, pedestals contain many other cytoskeletal modulators, including actin filament depolymerizing, filament cross-linking, membrane-tethering proteins as well as cytoskeletal motor proteins, myosins.^16^^,^^17^ For most of these actin-binding proteins, their roles in pedestal assembly and host cell colonization by A/E pathogens remain poorly understood.

One of the most obvious knowledge gaps is the lack of understanding of how non-muscle myosin II (NM II) controls A/E pathogen interactions with mammalian cells, particularly in the intestinal epithelium. NM II is a critical regulator of virtually all actin-based cellular processes and possesses a dual ability to translocate and cross-link actin filaments.^19^^,^^20^ IECs express two different NM II motors, NM IIA and NM IIC.^21^^,^^22^ These motors are enriched at the apical pole and intercellular junctions of enterocytes and regulate epithelial adhesion and microvilli dynamics.^21-23^ Several lines of indirect evidence suggest that NM II may play a role in A/E pathogen-induced remodeling of the actin cytoskeleton. First, immunofluorescence labeling and mass spectroscopy analysis detected NM II in EPEC pedestals.^24^^,^^25^ Second, the EPEC effector proteins EspB and Map are known to interact with NM II.^26^^,^^27^ Finally, EPEC and *C. rodentium* have been shown to stimulate NM II activity by increasing the phosphorylation of myosin light chains (MLC).^28-31^ While such NM II activation is thought to be essential for disrupting apical junctions in EPEC- and EHEC-infected intestinal epithelium,^30-32^ it remains unknown whether NM II controls pedestal assembly and bacterial attachment to host cells. This study was designed to fill this knowledge gap and examine the roles of NM II motors in regulating EPEC and *C. rodentium* interactions with the intestinal epithelium *in vitro* and *in vivo*. Our results highlight NM IIA as a unique negative regulator of A/E bacterial attachment to IECs, controlling bacteria-induced remodeling of the actin cytoskeleton and pedestal formation.

## Materials and Methods

### Antibodies and other reagents

The following primary polyclonal antibodies (pAb) and monoclonal antibodies (mAbs) were used to detect cytoskeletal proteins and EPEC: NM IIA pAb (BioLegend, San Diego, CA cat. #909801), LPS mAb (Abcam, Waltham, MA cat. #35654), NM IIC (D4A7) mAb (Cell Signaling, Beverly, MA cat. #8189S), and GAPDH (14C10) mAb (Cell Signaling, cat. #2118). Alexa Fluor-488- or Alexa Fluor-568-conjugated donkey anti-rabbit or donkey anti-mouse secondary antibodies, as well as F-actin probes, Alexa Fluor-488, Alexa Fluor-555, and Alexa Fluor-647-conjugate phalloidin, were obtained from Thermo Fisher Scientific (Waltham, MA). Horseradish peroxidase-conjugated goat anti-rabbit and anti-mouse secondary antibodies were purchased from Bio-Rad Laboratories (Hercules, CA). All other chemicals were obtained from Millipore-Sigma (St. Louis, MO).

### Animals

The mouse strain with intestinal epithelial-specific knockout of NM IIA was established by crossing NM IIA flox animals with constitutive Villin-Cre mice, as previously described.^22^ Transgenic NM IIA mouse strains with the replacement of the mouse NM IIA gene with the GFP-fused wild type human NM IIA or the R702C NM IIA mutant (referred to as GFP-NM IIA WT and GFP-NM IIA R702C, respectively) were described previously^33^^,^^34^ and were provided by Dr. Velia M. Fowler (University of Delaware). A mouse strain with total knockout of NM IIC (C57BL/6J-Myh14, NCBI ID 71960) was generated by (Cyagen, Santa Clara, CA) and provided by Dr. John Hammer (National Institutes of Health). The animal colonies were maintained under pathogen-free conditions with a 12 h light/dark cycle at the Cleveland Clinic vivarium. The mice were fed a standard chow diet of Envigo Diet 2918 (irradiated) and provided with filtered and chlorinated municipal water *ad libitum*. All procedures were performed as approved by the Cleveland Clinic Institutional Animal Care and Use Committee (IACUC protocol number 00001872) per the National Institutes of Health Animal Care and Use Guidelines.

### Bacterial strains and *in vitro* infection experiments

*Citrobacter rodentium* strain DBS100 was obtained from the American Type Culture Collection (ATCC, Manassas, VA. cat. #51459). The EPEC strain E2348/69 (Serotype O127:H6) was provided by Dr. Kaper for distribution by BEI Resources NIAID, NIH (cat. #NR-50518). EPEC mutant strains: EPEC-Δ*tir*, EPEC-Δ*tir* + pHA-Tir(WT), EPEC-Δ*tir* + pHA-Tir(Y454F/Y474F), and EPEC ΔT3SS were described previously.^35-38^

Single colonies of bacteria were propagated overnight by culturing in Luria-Bertani (LB) medium with agitation at 37 °C. The EPEC strains were subsequently subcultured for 3 h in the Dulbecco's minimum essential medium (DMEM) without antibiotics. The bacterial concentration was estimated by measuring the optical density at 600 nm, and the suspensions were diluted in their growth media to the desired MOI. For the *in vitro* experiments, epithelial cells were infected with EPEC at MOI 5:1 for 3 h. For immunolabeling experiments, EPEC infection was performed in confluent IEC monolayers growing on collagen-coated coverslips. For the CFU measurements, infection was performed in confluent IEC monolayers growing on collagen-coated 24-well plates. *C. rodentium* infection in mice is described below.

### Cell lines and generation of CRISPR/Cas9-mediated NM II knockout IECs

Caco-2BBE (cat. #CRL-2102) and Cos-7 (cat. #CRL-1651) were purchased from ATCC. HT-29cF8, a well-differentiated clone of HT-29 cells^39^^,^^40^, was provided by Dr. Judith M. Ball (College of Veterinary and Biomedical Sciences, Texas A&M University, College Station, TX). HT-29, Caco-2, and Cos-7 cells were cultured in DMEM medium supplemented with 10% fetal bovine serum (FBS), HEPES, non-essential amino acids, and penicillin‒streptomycin antibiotics. NM IIA and NM IIC knockout in IEC lines was achieved using the CRISPR/Cas9 V2 system as we described previously.^39^ Single guide RNAs (sgRNAs) specific for *NM IIA* or *NM IIC* were designed via the CRISPR design tool developed by the Feng Zhang Laboratory (http://crispr.mit.edu, McGovern Institute, MIT, Boston, MA, USA). The sgRNA sequences used were: NMIIA sg1 5′-ACGCCACGTACGCCAGATAC and NMIIA sg8 5′-CTGAGTAGTAACGCTCCTTG and NM IIC sg1 5′-CCTCGGTCACTGCGTACACG, and NM IIC sg3 5′-GATTACTCACGTGCAGAGAA. Each sgRNA was cloned into the BbsI site of the lenti-CRISPR v2 vector (Addgene, Watertown, MA, cat. #52961), with successful insertion confirmed by sequencing. Lentiviral particles were produced in HEK293T cells co-transfected with the lenti-CRISPR-sgRNA plasmids and packaging plasmids pCD/NL-BH*DDD (Addgene cat. #17531) and pLTR-G (Addgene cat. #17532), using the TransIT-293 transfection reagent (Mirus Bio, Madison, WI, USA). To establish stable knockouts, HT-29 and Caco-2 BBE cells were transduced with the resulting lentiviruses and subjected to puromycin selection (5 μg/mL) for 7 d. Control cells were generated by transduction with a lentivirus carrying a non-targeting sgRNA, followed by the same selection protocol. For bacterial infections, HT-29cF8 and Caco-2BBE cells were cultured in collagen-coated 24-well plates or on coverslips for 7 and 14 d, respectively, to allow the formation of confluent differentiated epithelial cell monolayers.

### Generation and expression of human NM IIA mutants

To generate NM II mutants, the backbone plasmid CMV-GFP-NM IIA, in which the human *NM IIA* gene is fused to GFP (Addgene, cat. #11347), was used. Individual point mutations N93K and R702C were introduced using the QuikChange Lightning site-directed mutagenesis kit (Agilent Technologies, Santa Clara, CA, cat. #200515) as we recently described.^41^ Briefly, the phosphorylated primers 5′-GCACCGAGGCTTCCTTGAGGCACGTGA (N93K) and 5′-CTGGCGGCAGATACAGATGCCCTCGAGAA (R702C) were used in the PCR-based mutagenesis reaction, and the obtained PCR products were digested with the restriction enzyme DpnI. The resulting reaction mixtures were transformed into Agilent Technologies XL10-Gold® ultracompetent cells (Agilent Technologies, Santa Clara, CA, cat. #200314). Plasmid DNA was isolated from individual colonies using the PureYield Miniprep System (Promega, Madison, WI, cat. #A1222). The presence of the desired mutations was verified by DNA sequencing. For transfection, Cos-7 cells were seeded onto collagen-coated coverslips at 50% confluence and transfected the following day with the indicated plasmids at a final concentration of 0.5 µg/mL using the TransIT-2020 transfection reagent (Mirus Bio, Madison, WI, cat. #MIR 5404) according to the manufacturer's instructions. Twenty-four hours post-transfection, the cells were infected with EPEC at MOI 5:1 for 3 h. Unattached bacteria were removed by thorough washing with PBS, and the cells were subjected to immunofluorescence labeling and confocal microscopy as described below.

### Induction and characterization of *C. rodentium* infection

All experiments involving *C. rodentium* infection were performed in an ABSL-2 level facility following an established procedure.^42^ Both male and female mice, aged 8–10 weeks, were used for the experiments, with approximately equal distributions of sexes across different experimental groups. The animals were given a streptomycin solution (4 g/L) in the drinking water for 24 h, followed by 24-h exposure to normal tap water and overnight food deprivation before bacterial gavage. A stationary phase *C. rodentium* culture was prepared by overnight culture in liquid LB, and the concentration of the bacterial suspension was determined by measuring its optical density at 600 nm. Animals were gavaged with 200 µL of the bacterial suspension containing 1 × 10^9^ colony-forming units per mouse or 200 µL of LB medium as a control. The day of gavage was designated as day 0. The mice were monitored daily for clinical signs of infection, including weight loss, reduced activity, and changes in stool consistency.

To assess bacterial colonization, fecal samples were collected every other day post-infection. Fecal pellets were collected directly into pre-weighed sterile microcentrifuge tubes containing 0.5 ml of PBS to allow normalization of the bacterial load to the sample mass. Furthermore, after each experiment, approximately 1 cm segments of distal colon and ileum, as well as 0.5 cm segments of cecum tissue, were excised, washed with sterile phosphate-buffered saline (PBS), and placed into pre-weighed sterile tubes.

The samples were homogenized in sterile PBS using a homogenizer with disposable blades ensuring thorough disruption of the tissue and release of bacteria. The homogenates were subjected to ten-fold serial dilutions in sterile PBS, with aliquots of each dilution plated on MacConkey agar plates in triplicate. The plates were incubated at 37 °C for 16–18 h, after which pink lactose-fermenting colonies characteristic of *C. rodentium*, were counted. The bacterial colony counts were used to calculate CFUs per gram of feces or intestinal tissue, allowing quantitative assessment of the bacterial burden.

### Measurement of epithelial barrier permeability *in vivo*

Intestinal permeability in NM IIA cKO mice and their control, flox littermates was measured as previously described.^22^^,^^43^ Briefly, the animals were placed into new cages without bedding and subjected to 3 h of food deprivation. Each animal received fluorescein isothiocyanate (FITC)-labeled dextran (4 kDa. Millipore Sigma, cat. #78331) dissolved in phosphate-buffered saline (PBS) at a dose of 80 mg/100 g body weight via oral gavage. The animals were euthanized 3 h after FITC-dextran administration for blood collection via cardiac puncture into vacutainer tubes with anticoagulant. Blood plasma was obtained by centrifugation, and the FITC fluorescence intensity was measured using a Synergy H1 microplate reader (Agilent Technology) with excitation and emission wavelengths at 495/525 nm. The measured value of dextran-free serum was subtracted from each measurement. The concentration of the fluorescent tracer in blood plasma was calculated using SigmaPlot v12.5 software, using a standard curve prepared via serial dilutions of stock solutions of FITC-dextran in PBS.

### Immunoblotting analysis

Immunoblotting analysis was performed as we previously described.^22^^,^^41^^,^^43^ Briefly, mouse colonic segments were longitudinally dissected, opened, and washed with ice-cold PBS. The epithelial cells were collected by gently scraping the exposed interior with razor blades, then snap frozen into liquid nitrogen for further analysis. Intestinal epithelial scrapings were homogenized in a radioimmunoprecipitation assay (RIPA) lysis buffer containing a protease inhibitor cocktail, phosphatase inhibitor cocktails 2 and 3, and Pefabloc (all from Millipore-Sigma). The samples were diluted 1:1 with 2× SDS sample loading buffer and boiled. Cultured colonic epithelial cell monolayers were processed in a similar way using the RIPA buffer and 2× SDS sample buffers. SDS‒polyacrylamide gel electrophoresis of tissue scraping and cell lysates was conducted using a standard protocol with an equal amount of total protein loaded per lane (10 µg), followed by overnight protein transfer to a nitrocellulose membrane. The membranes were blocked with a blocking buffer containing 5% non-fat milk and sequentially incubated with primary antibodies (at 1:1000 dilution) and secondary horseradish peroxidase-conjugated antibodies (at 1:10,000 dilution). The labeled membranes were exposed to enhanced chemiluminescence reagent (Millipore-Sigma) with the chemiluminescent signals were captured on X-ray film using an automated film processor. Signal intensities were quantified via densitometry using ImageJ 1.51K software (National Institutes of Health, Bethesda, MD).

### Quantitative real-time RT-PCR

The quantitative RT-PCR analysis of different inflammatory mediators was performed according to previously published procedures.^22^^,^^43^ Briefly, total RNA was isolated from whole colonic segments of NM IIA cKO mice and their control littermates using a RNeasy Mini Kit (Qiagen, Germantown, MD, cat. #74104) and was reverse transcribed using an iScript cDNA synthesis kit (Bio-Rad Laboratories, cat. #1708891). Quantitative real-time RT-PCR was performed using iTaq Universal SYBR Green Supermix (Bio-Rad Laboratories, cat. #1725121) in a CFX96 real-time PCR system (Bio-Rad Laboratories) using previously published primer sequences.^22^ The threshold cycle number (Ct) for specific genes of interest and a housekeeping gene was determined based on the amplification curves. The relative expression of each gene was calculated by a comparative Ct method based on the inverse proportionality between Ct and the initial template concentration (2^−^^ΔΔCt^), as previously described.^44^ This method is based on two-step calculations of ΔCt = Ct_target gene_ − Ct_GAPDH_ and ΔΔCt = ΔCt_e_ − ΔCt_c_, where the index e refers to the sample from any *C. rodentium* or LB-treated NM IIA cKO, or control mice, and index c refers to the sample from a LB-treated control animal assigned as an internal control.

### Immunofluorescence labeling, confocal microscopy, and image analysis

Bacterial attachment and the organization of the actomyosin cytoskeleton in HT-29cF8 and Caco-2BBE cell monolayers were determined using immunofluorescence labeling and confocal microscopy, as we previously described.^39^^,^^41^^,^^45^ Briefly, the cells were fixed/permeabilized either at room temperature with 4% paraformaldehyde and 0.5% Triton X-100 solutions or at −20 °C with 100% ethanol. Fixed cells were blocked for 60 min at room temperature with 1% bovine serum albumin (BSA) dissolved in Hank's balanced salt solution (HBSS+). The blocked cells were sequentially incubated with primary antibodies diluted in blocking buffer, washed, and incubated with Alexa Fluor–conjugated secondary antibodies, rinsed with blocking buffer, and mounted on slides using ProLong™ Gold Antifade Reagent (Thermo Fisher Scientific, cat. #P36930). The actin filaments and nuclei were labeled with Alexa Fluor-conjugated phalloidin and 4′,6-diamidino-2-phenylindole (DAPI), respectively.

Fluorescently labeled monolayers were imaged using 63× or 100× oil immersion objectives (1.4 NA) on a laser scanning confocal microscope (DMi8 inverted microscope equipped with TCS SP8 confocal scanner (Leica Microsystems, Wentzler, Germany), using LAS-X acquisition software (Leica Microsystems, v3.5.5). Actin pedestals along the cell‒bacteria interface were visualized by using LAS-X 3D (v4.8.0) to segment 3D volumes reconstructed from *z*-stacks acquired with lateral (XY) and axial (XZ) resolutions of 0.07 and 0.15 micron/pixel, respectively. We also acquired confocal images in XYZ mode to capture cross-sections at the bacterial‒pedestal interaction sites, followed by the extraction of pixel intensity values using LAS-X to generate a line profile plot illustrating overlap between bacteria, NM II paralogs and F-actin.

Individual bacteria or bacterial colonies were quantified using ImageJ. The particle size thresholds were set to 10 pixels² for individual bacteria in the case of HT-29 cells and 80 pixels² for bacterial colonies associated with Caco-2 cells. To quantify bacterial attachment, the bacterial count in six different microscopic fields per coverslip was averaged to produce a single data point. Three different coverslips per experimental condition were examined, and the data obtained in two or three different experiments were combined and analyzed. The numbers of biological replicated obtained in the combined experiments are presented in the Figure legends.

F-actin and NM IIA images were processed and analyzed using Volocity (v6.5.1, Quorum Technologies Inc., Puslinch, ON, Canada). A median filter was first used to suppress noise contributions from the raw data before colocalization calculations were performed. For each condition, the total stress fiber intensity was quantified by the pixel intensities of all visible stress fibers in 4–6 images per group and computing the average. To demonstrate the colocalization of the NM IIA and F-actin signals, both Pearson's correlation coefficient and Mander's overlap coefficient were then calculated to define the spatial relationship between NM IIA and F-actin. Pearson coefficient takes into account the intensity of the marker in addition to the overlapping signal, whereas Mander's coefficient calculates the overlap between M1 and M2. M1 means percentage of pixels from Channel 1 that overlaps with Channel 2 and M2 means percentage of Channel 2 pixels that overlaps with Channel 1.

### Bacterial colony forming assay

Confluent differentiated IEC monolayers cultured in 24 well plates were infected with EPEC at a multiplicity of infection (MOI) of 5:1. The cells were co-cultured with bacteria in DMEM without antibiotics for 3 h and then washed three times with PBS to remove non-adherent bacteria. Next, the cells were lysed on ice using 200 µL per well of 1% Triton X-100 in PBS for 10 min. The lysates from each well were transferred to microcentrifuge tubes and vortexed. Serial dilutions of the lysates were then plated onto agar plates, which were incubated overnight at 37 °C. The next morning, bacterial colonies were counted, and the total number of attached bacteria was calculated as colony forming units per well. Bacterial attachment to three different cell monolayers was counted for each experimental condition. Data combined from 2–3 independent experiments were analyzed for statistically significant differences.

### Statistics

All numerical data are presented as a mean ± SEM. The statistical significance of the difference between the two sets of data was evaluated using the two-tailed unpaired Student's *t*-test when the data were distributed normally. Differences in body weight loss and bacterial fecal content data were tested for statistical significance using one-way ANOVA (SigmaPlot 12.5 package) with Tukey post-hoc test. Statistical significance was accepted at *p* < 0.05.

## Results

### Mice with intestinal epithelium-specific knockout of NM IIA show increased susceptibility to *C. rodentium* infection

**Figure 1.**
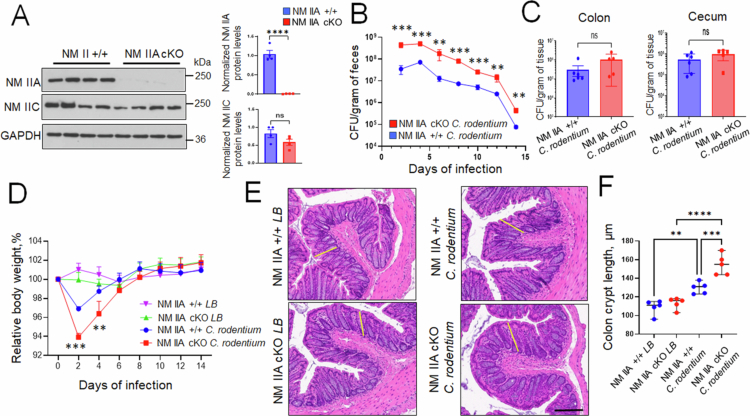
Loss of the intestinal epithelial NM IIA accelerates *Citrobacter rodentium* infection in mice. (A) Immunoblotting analysis and densitometric quantification of NM IIA and NM IIC expression in the colonic epithelial scrapes of NM IIA cKO and flox control (NM IIA+/+) mice. (B–F) Control and NM IIA cKO mice were gavaged with *C. rodentium* at 1 × 10^9^ CFU per mouse. (B) Bacterial shedding in the fecal pellets was examined at the indicated times of infection. (C) Bacterial colonization of the colon and cecum was determined on day 14 of infection. (D) The change in the body weight of the control and *C. rodentium*-infected animals was determined at different times after bacterial administration. (E and F) Crypt length (indicated by yellow bars) was measured in H&E-stained colonic sections of control and *C. rodentium*-infected mice on day 14 after bacterial administration. Mean ± SEM, *n* = 4–6; **p < 0.01, ***p < 0.001, ****p < 0.0001; ns, not significant; scale bar = 200 μm.

Intestinal epithelial cells are known to express two NM II motors, NM IIA and NM IIC, with NM IIA being crucial for establishing the protective intestinal epithelial barrier and limiting mucosal inflammation.^22^^,^^46^ To examine the roles of this cytoskeletal motor in regulating the colonization of the intestinal epithelium by A/E pathogens *in vivo*, we infected mice with an intestinal epithelial-specific knockout of NM IIA (NM IIA cKO) with *Citrobacter rodentium*. NM IIA flox littermates (referred to as NM IIA+/+) served as controls. In agreement with our previous data,^22^ NM IIA cKO mice displayed a selective loss of NM IIA protein expression in the intestinal epithelium without altered expression of NM IIC motor (Figure 1A). NM IIA cKO and control mice were infected with *C. rodentium* at 1 × 10^9^ CFU per mouse, and bacterial colonization was determined by measuring viable *C. rodentium* in fecal pellets by an agar colony forming assay. NM IIA cKO mice demonstrated significantly higher bacterial loads on days 2–14 after *C. rodentium* administration as compared to the control animals (Figure 1B). Despite such higher initial colonization in NM II cKO mice, the rate of bacterial clearance appears to be similar between the two mouse strains (Figure 1B). Consistently, two weeks post-infection, the amounts of *C. rodentium* recovered from cecal and colonic tissues were not different between NM IIA cKO and their flox littermates, indicating that both animal strains can efficiently clear the pathogen from the intestine (Figure 1C). Interestingly, NM II cKO mice demonstrated exaggerated pathophysiologic responses to bacterial infection. These include enhanced body weight loss in knockout mice compared to the control flox littermates at the early phase of infection (Figure 1D) and more pronounced colonic crypt hyperplasia following two weeks of *C. rodentium* infection (Figure 1E and F).

**Figure 2.**
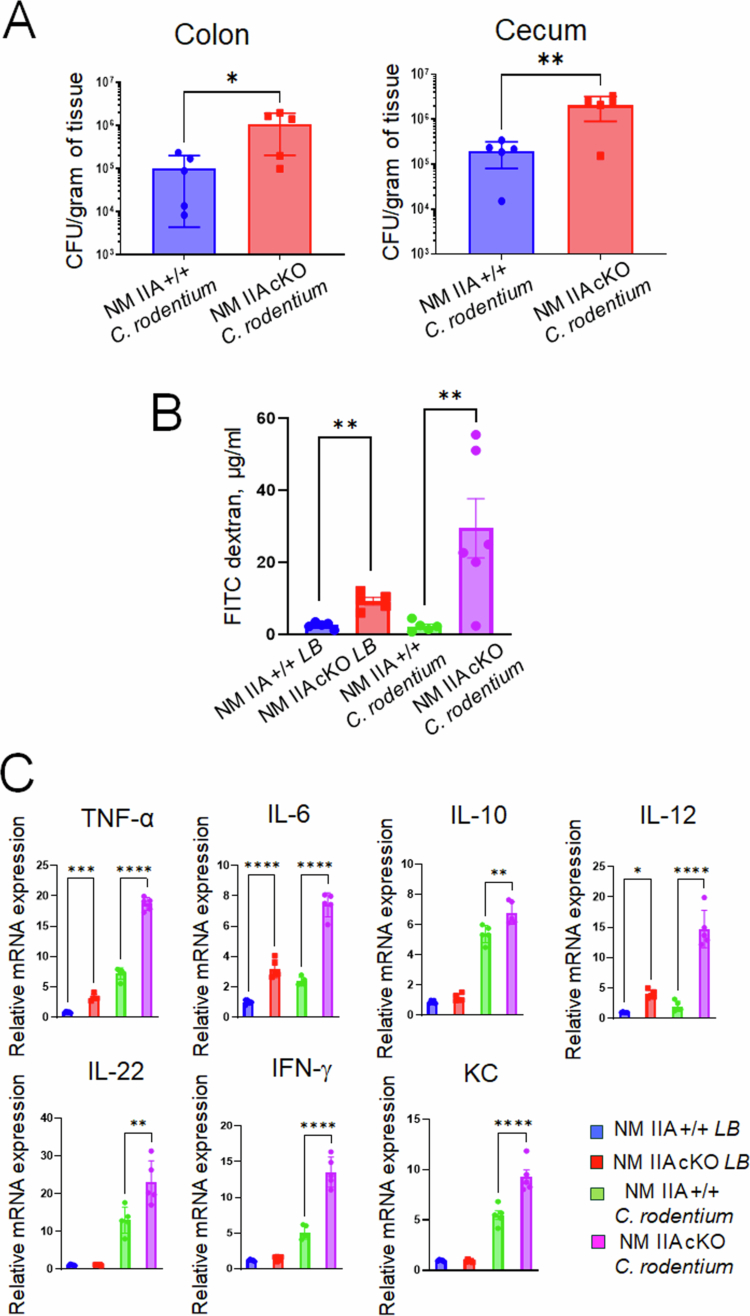
Intestinal epithelial specific knockout of NM IIA increases animal sensitivity to *C. rodentium*-induced gut barrier disruption and mucosal inflammation. Control and NM IIA cKO mice were gavaged with *C. rodentium* at 1 × 10^9^ CFU per mouse. (A) Bacterial colonization of the colon and cecum was determined on day 8 of infection. Mean ± SEM, *n* = 5; *p < 0.05, **p < 0.01. (B) The gut-to-blood passage of 4 kDa FITC-dextran was measured in NM IIA cKO and control mice with and without *C. rodentium* infection. Mean ± SEM, *n* = 5–6; **p < 0.01. (C) mRNA expression of inflammatory markers was measured in the colonic tissues of NM IIA cKO and control mice on day 8 of *C. rodentium* infection. Mean ± SEM, *n* = 5; *p < 0.05, **p < 0.01 *** *p* < 0.001, and ****p < 0.0001.

Next, we sought to characterize *C. rodentium*-induced intestinal inflammation early post-infection (8 d). In this shorter infection experiment, we observed an approximately 1log increase in bacterial load in both fecal samples (Supplementary Figure 1) as well as in the colonic and cecal tissues of NM IIA cKO mice (Figure 2A), indicating that loss of NM IIA enhances intestinal colonization by the pathogen. Since A/E bacteria are known to induce breakdown of the intestinal epithelial barrier,^31^^,^^32^ we examined gut barrier integrity in control and NM IIA cKO mice with and without *C. rodentium* infection. Consistent with our previously published data,^22^ NM IIA cKO mice demonstrated increased intestinal permeability under basal conditions (Figure 2B). Furthermore, *C. rodentium* infection triggered further disruption of the gut barrier in NM IIA cKO mice without affecting barrier integrity in control animals (Figure 2B). Next, we evaluated pathogen-induced mucosal inflammation by measuring the mRNA expression of major proinflammatory mediators in colonic tissue samples of NM IIA cKO and control animals. Even unchallenged NM IIA cKO mice showed significant increases in the tissue expression of TNFα and interleukins (ILs)-6 and -12, which may suggest a low level of spontaneous inflammation in their intestine (Figure 2C). *C. rodentium* infection resulted in significantly higher mRNA expression of TNFα, IL-6, IL-10, IL-12, IL-22, interferon-γ, and keratinocyte factor chemokine (KC) in the colonic tissues of NM IIA cKO mice as compared to their flox controls (Figure 2C). Together, our data demonstrate that the loss of intestinal epithelial NM IIA increases susceptibility to *C. rodentium* infection and promotes pathogen-induced disruption of the gut barrier, mucosal inflammation, and colonic crypt hyperplasia *in vivo*.

### Pharmacologic or genetic inhibition of NM IIA motor accelerates EPEC attachment to intestinal epithelial cell monolayers in vitro

**Figure 3.**
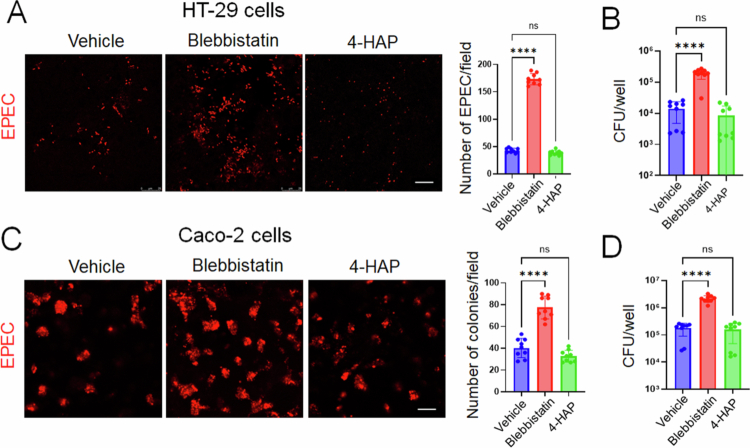
Pharmacological inhibition of NM II motor activity promotes EPEC attachment to model intestinal epithelial cell monolayers *in vitro*. Confluent differentiated HT-29cF8 (A and B) Caco-2BBE (C and D) cell monolayers were exposed to EPEC (MOI 5:1) for 3 h in the presence of either vehicle, blebbistatin (50 µM) or 4-HAP (100 µM). Bacterial attachment to IEC was determined by either immunofluorescence labeling/confocal microscopy of LPS (A and C) or a colony forming assay (B and D). Mean ± SEM of combined data from three independent experiments, *n* = 9; ****p < 0.001; scale bar = 20 μm.

The increased sensitivity of NM IIA cKO mice to *C. rodentium* infection could be due to enhanced attachment of the A/E pathogen to the NM IIA-depleted intestinal epithelium or priming effects of gut barrier disruption and the low-scale mucosal inflammation observed in NM IIA cKO mice (Figure 2). To distinguish between these two possibilities, we adopted a reductionistic *in vitro* approach to examine the attachment of a key human A/E bacterium, EPEC, to HT-29cF8, and Caco-2BBE human colonic epithelial cells. The interactions of EPEC with confluent, differentiated IEC monolayers were examined by either visualizing attached bacteria with immunofluorescence labeling for LPS and confocal microscopy or by quantifying live bacteria attached to monolayers by colony-forming assay. NM II motor activity was blocked by a specific pan-NM II inhibitor, blebbistatin.^47^ This was complemented by a pharmacologic gain-of-function approach, utilizing 4-hydroxyacetophenone (4-HAP) that is known to selectively activate NM IIB and NM IIC activity without affecting NM IIA.^48^ Differentiated IEC monolayers were preincubated for 1 h with either blebbistatin, 4-HAP or vehicle (DMSO) followed by 3 h EPEC infection in the presence of the indicated compounds or vehicle. Blebbistatin treatment significantly increased EPEC attachment to HT-29 (Figure 3A and B) and Caco-2 (Figure 3C and D) cells in both adhesion assays. By contrast, 4-HAP treatment did not significantly change EPEC interactions with IECs (Figure 3).

**Figure 4.**
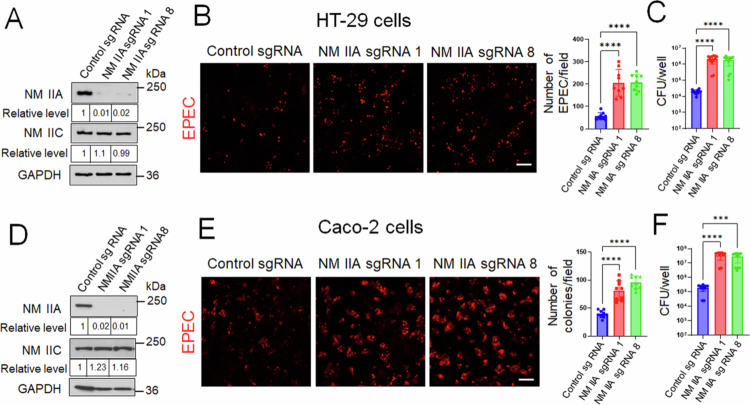
CRISPR-Cas9-mediated knockout of NM IIA promotes EPEC attachment to model intestinal epithelial cell monolayers *in vitro*. (A and D) Immunoblotting analysis of NM IIA and NM IIC expression in HT-29cF8 and Caco-2BBE cells with CRISPR/Cas9-mediated knockout of NM IIA using two different sgRNAs. (B–E). Control and NM IIA knockout HT-29 (B and C) and Caco-2 (E and F) cells were infected with EPEC (MOI 5:1) for 3 h. Bacterial attachment to IEC was determined by either immunofluorescence labeling/confocal microscopy of LPS (B and E) or colony forming assay (C and F). Mean ± SEM of combined data from three independent experiments, *n* = 9; ***p < 0.001, ****p < 0.0001; scale bar = 20 μm.

Next, we examined whether NM IIA is required for restricting EPEC attachment to model IEC monolayers. To examine the functional role of this cytoskeletal motor, we created IEC lines with stable CRISPR/Cas9-induced knockout of NM IIA. Using two different sgRNAs, we achieved a marked reduction (up to 99%) of NM IIA protein expression in HT-29 and Caco-2 cells without affecting NM IIC levels (Figure 4A and D). Such NM IIA knockout significantly upregulated EPEC attachment in both HT-29 (Figure 4B and C) and Caco-2 cell monolayers (Figure 4D and E), thereby recapitulating the results obtained with *C. rodentium* infection of NM IIA cKO mice.

### Loss of NM IIC expression does not affect A/E bacterial interactions with intestinal epithelial cells in vitro and in vivo

**Figure 5.**
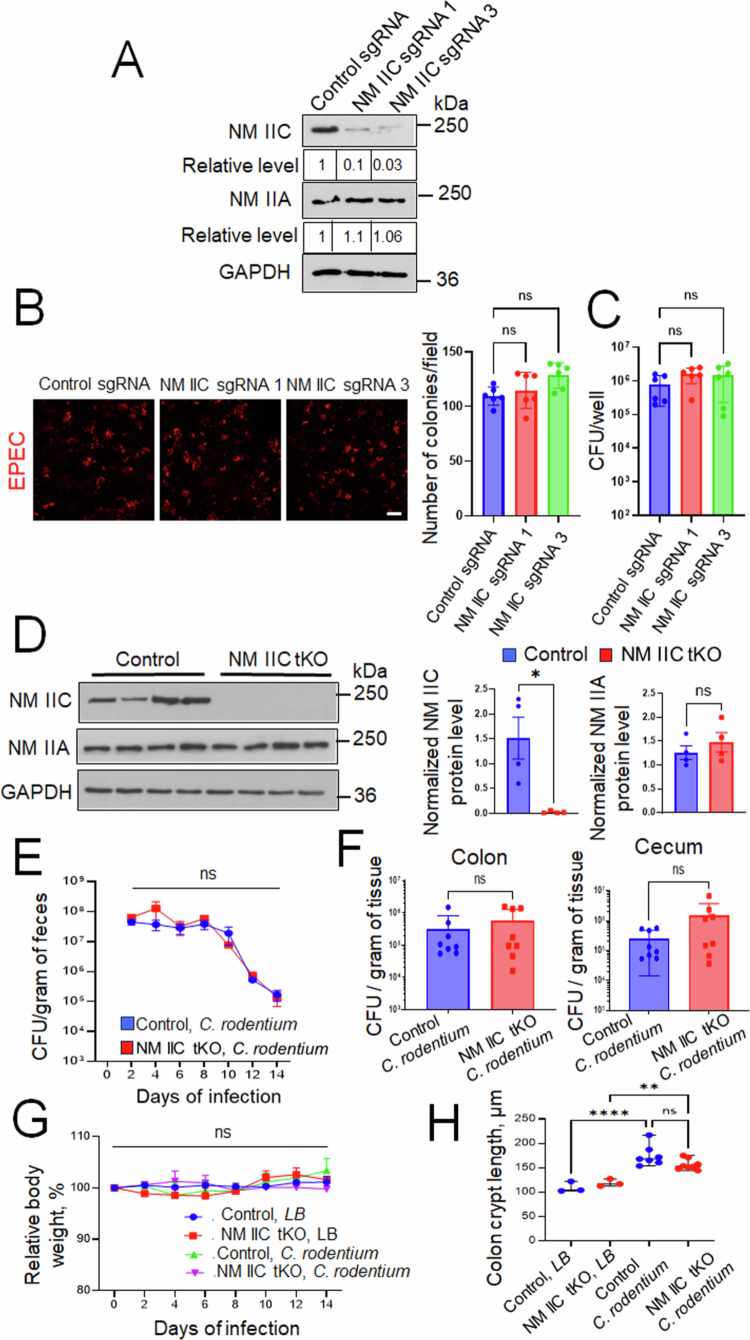
Loss of intestinal epithelial NM IIC does not affect A/E bacterial interactions with the intestinal epithelium *in vitro* and *in vivo*. (A) Immunoblotting analysis of NM IIC and NM IIA expression in Caco-2BBE cells with CRISPR/Cas9-mediated knockout of NM IIC using two different sgRNAs. (B and C) Control and NM IIC knockout Caco-2 cells were infected with EPEC (MOI 5:1) for 3 h. Bacterial attachment to IECs was determined by either immunofluorescence labeling/confocal microscopy of LPS (B) or a colony-forming assay (C). Mean ± SEM of combined data from two independent experiments, *n* = 6; ns, not significant. (D) Immunoblotting analysis of NM IIC and NM IIA expression in the colonic epithelial scrapes of NM IIC knockout and control mice. Mean ± SEM, *n* = 4; *p < 0.05 (E–H) Control and NM IIC KO mice were gavaged with *C. rodentium* at 1 × 10^9^ CFU per mouse. (E) Bacterial shedding in fecal pellets was examined at the indicated times of infection. (F) Bacterial colonization of the colon and cecum was determined on day 14 of infection. (G) The change in the body weight of the control and *C. rodentium*-infected animals determined over time. Mean ± SEM, *n* = 8. (H) Colonic crypt length was measured in the control and *C. rodentium*-infected mice on day 14 after bacterial administration. Mean ± SEM, *n* = 3–8; **p < 0.001, **** *p* < 0.0001; scale bar = 20 μm.

Next, we sought to investigate if another major IEC actin motor, NM IIC, plays role in mediating A/E pathogens interactions with the intestinal epithelium. CRISPR/Cas9-mediated knockout of NM IIC caused a significant decrease in its protein level in Caco-2 cells, while NM IIA expression remained unaffected (Figure 5A). However, unlike NM IIA knockout, loss of NM IIC did not impact EPEC attachment to IEC monolayers, as shown by immunofluorescence labeling/confocal microscopy of attached bacteria (Figure 5B) or bacterial colony-forming assays (Figure 5C). We sought to determine the *in vivo* relevance of this finding by examining *C. rodentium* infection in mice with total knockout of NM IIC (NM IIC tKO). Predictably, NM IIC tKO mice showed complete loss of NM IIC protein expression in the colonic epithelium, while NM IIA levels remained unchanged (Figure 5D). Both NM IIC tKO and control mice demonstrated similar responses to *C. rodentium* infection, based on their similar bacterial loads in feces and intestinal tissues (Figure 5E and F), lack of significant body weight loss (Figure 5G), and have similar levels of colonic crypt hyperplasia (Figure 5H). Together, these data suggest that the NM IIC motor does not regulate interactions of A/E pathogens with the intestinal mucosa *in vivo* and *in vitro*.

### Motor domain activity is essential for NM IIA-dependent regulation of A/E bacteria interactions with the intestinal epithelium

**Figure 6.**
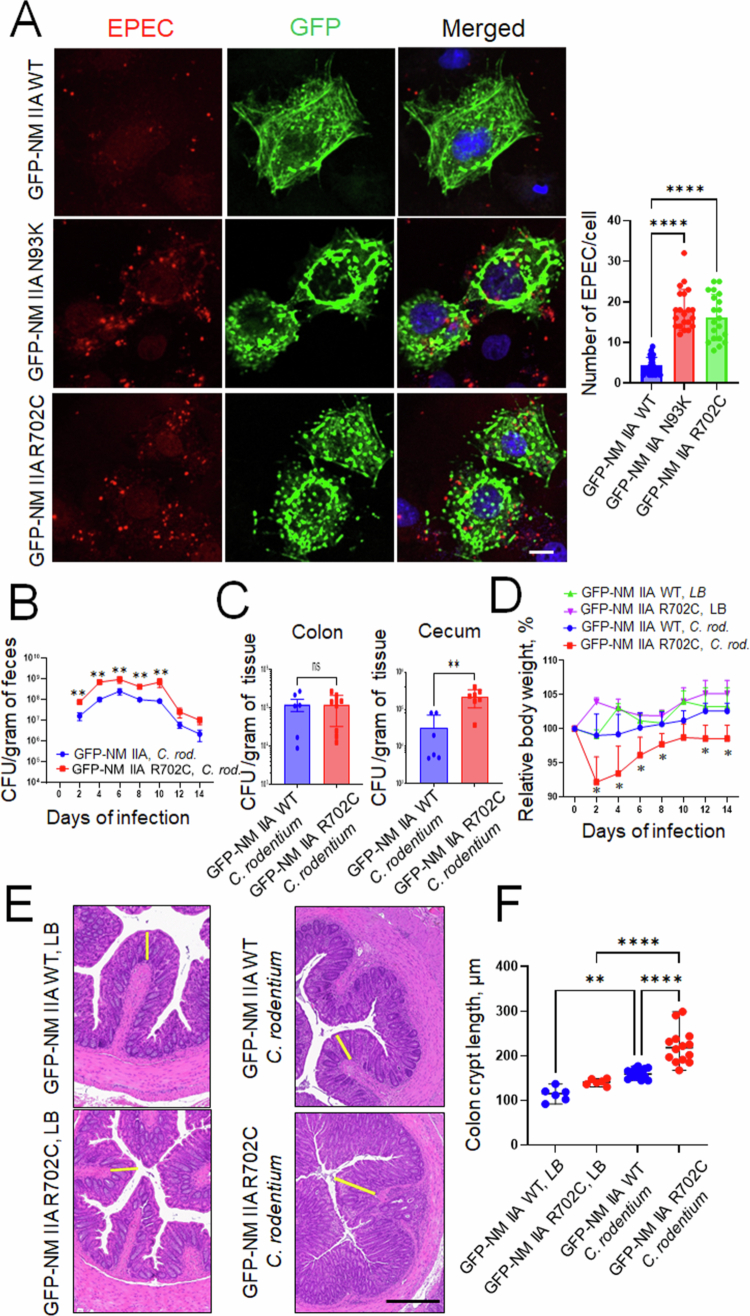
Mutations in the motor domain of NM IIA promote A/E bacterial interactions with the intestinal epithelium *in vitro* and *in vivo*. (A) EPEC attachment to Cos-7 cells (MOI 5:1 for 3 h) transfected with GFP-labeled wild-type (WT) NM IIA or GFP-labeled NM IIA R702C and N93K mutants was determined by immunofluorescence labeling and confocal microscopy. Mean ± SEM, *n* = 20–25 individual cells per group, ****p < 0.0001; scale bar = 10 μm. Data is representative of three independent experiments. (B–F) Transgenic mice expressing either the GFP-NM IIA WT or the GFP-NM IIA R702C mutant were gavaged with *C. rodentium* at 1 × 10^9^ CFU per mouse. (B) Bacterial shedding in the fecal pellets was examined at the indicated times of infection. (C) Bacterial colonization of the colon and cecum was determined on day 14 of infection. (D) The body weight of the control and *C. rodentium*-infected animals was measured at different times after bacterial administration. Mean ± SEM, *n* = 3–6; *p < 0.05; **p < 0.01. (E and F) Colonic crypt length was measured in the control and *C. rodentium*-infected mice on day 14 after bacterial administration. Mean ± SEM, *n* = 6–12; **p < 0.001, **** *p* < 0.0001; scale bar = 300 μm.

Given the observed unique roles of NM IIA in limiting A/E bacterial attachment to IECs, we next sought to understand what properties of this cytoskeletal protein are essential for controlling host‒bacterial interactions. Like other conventional myosins, NM IIA has dual functionality as an actin motor and cross-linking protein.^19^^,^^20^ Its motor function is determined by the N-terminal globular domain that interacts with actin filaments and possesses ATPase activity.^19^^,^^20^ Several point mutations in the N-terminal domain were shown to inhibit the motor activity of NM II.^49^^,^^50^ We examined whether the motor activity of NM IIA is essential for controlling A/E bacterial interactions with mammalian cells by utilizing two motor domain mutations, R702C and N93K. The GFP-tagged mutants, along with wild-type NM IIA, were transiently expressed in Cos-7 epithelial cells that lack endogenous NM IIA. EPEC interactions with GFP-NM IIA-expressing cells were determined by immunolabeling and confocal microscopy. Bacterial attachment to Cos-7 cells expressing GFP-NM IIA mutants was significantly higher than attachment to wild-type GFP-NM IIA-expressing cells (Figure 6A). To establish the *in vivo* relevance of this finding, we used transgenic mouse strains expressing GFP-labeled WT human NM IIA or its R702C mutant under the control of the endogenous mouse promoter.^34^ GFP-NM IIA WT and GFP-NM IIA R702C mice were infected with *C. rodentium* for 14 d, and the bacterial infection was characterized as described above. Compared to GFP-NM II WT animals, the bacteria-infected GFP-NM IIA R702C mice demonstrated approximately ten fold higher fecal bacterial loads during infection (Figure 6B) and significantly elevated *C. rodentium* levels in cecal tissue 14 d after bacterial administration (Figure 6C). Furthermore, the mutant mice also displayed more pronounced body weight loss (Figure 6D) and colonic crypt hyperplasia compared to GFP-NM IIA WT controls (Figure 6E and F), thereby recapitulating higher sensitivity to *C. rodentium* infection observed in the NM IIA cKO mice. Collectively, these results indicate that the motor activity of NM IIA is essential for limiting A/E bacterial interactions with the intestinal epithelium *in vivo* and *in vitro*.

### NM IIA controls Tir-dependent EPEC attachment to IEC monolayers

**Figure 7.**
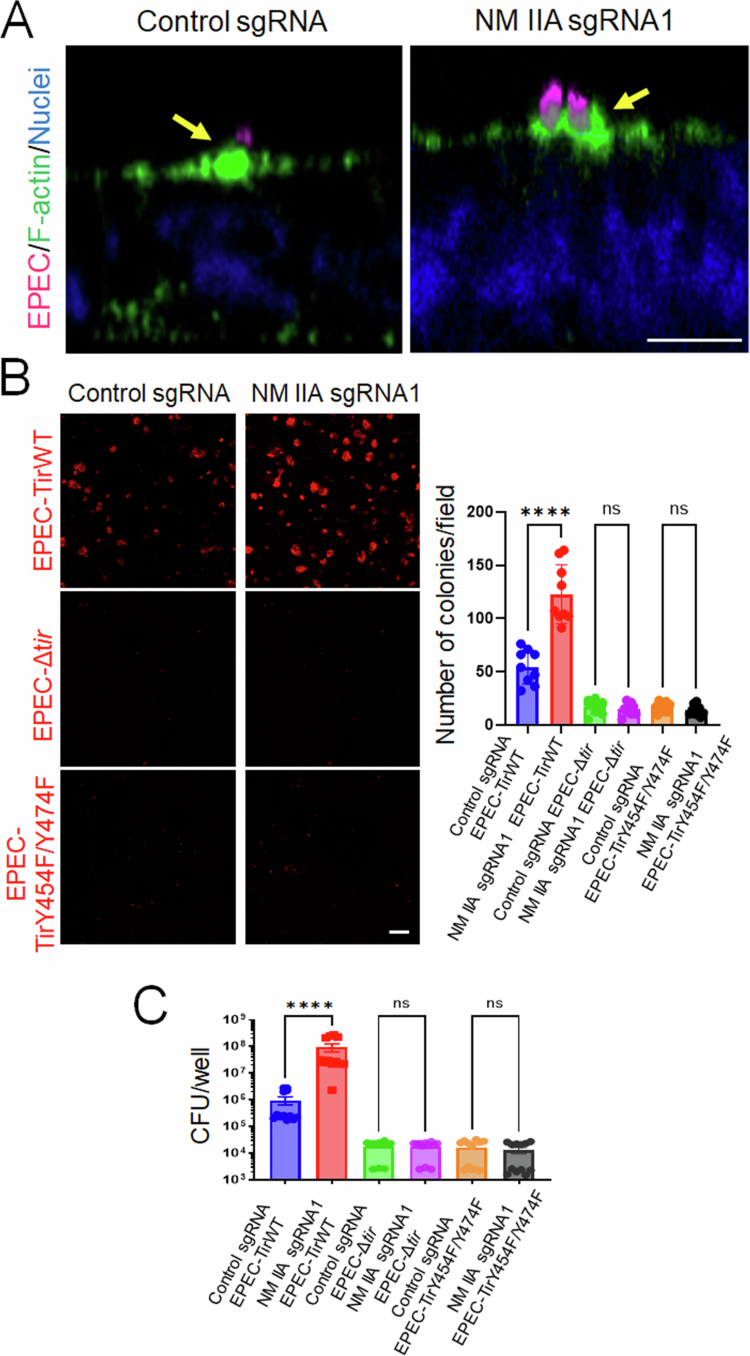
NM IIA does not affect the attachment of pedestal assembly-deficient EPEC mutants to IECs. (A) Control and NM IIA knockout HT-29cF8 cells were infected with EPEC (MOI 5:1) for 3 h. The cells were fixed and fluorescently labeled for F-actin (green) and bacteria (red). Arrows point at F-actin pedestals under bacterial colonies in control and NM-IIA knockout cells. Scale bar = 10 μm. (B and C). Control and NM IIA-knockout HT-29 cells were infected for 3 h with either a Tir-deficient EPEC strain (EPEC-Δ*tir*) or a bacterial strain reconstituted with wild-type Tir (EPEC-TirWT) or its Y454F/Y474F mutant (EPEC-TirY454F/Y474F). Bacterial attachment to IECs was determined by either immunofluorescence labeling/confocal microscopy of LPS (B) or a colony-forming assay (C). Mean ± SEM of combined data from three independent experiments, *n* = 9; ****p < 0.0001; scale bar = 20 μm.

Next, we sought to investigate the mechanisms by which the NM IIA motor attenuates the attachment of A/E bacteria to the intestinal epithelium. We rationalized that NM II most likely regulates the actin cytoskeletal remodeling induced by bacterial attachment, more specifically, the assembly of F-actin pedestals. Indeed, fluorescence labeling revealed the formation of prominent F-actin pedestals at EPEC attachment sites in both control and NM IIA-deficient IEC monolayers (Figure 7A, arrows). To decisively prove that NM IIA controls EPEC attachment to IECs in an actin pedestal assembly-dependent fashion, we investigated cellular interactions with EPEC mutants that lack a key actin pedestal-forming effector, Tir.^51^ Specifically, we compared cell attachment of the bacterial strain with total Tir deletion (EPEC-Δ*tir*) and EPEC strains reconstituted with either wild-type Tir (EPEC-TirWT) or with mutations of two tyrosine residues, Y454F and Y474F, which are responsible for triggering actin polymerization and pedestal assembly (EPEC-TirY454F/Y474F).^35-38^ As an additional control, we used an EPEC mutant with a deleted Type III secretion system that is unable to introduce any bacterial effectors into the host cells.^37^^,^^38^ As expected, the infection of HT-29 cells monolayers with the EPEC-TirWT strain resulted in robust assembly of F-actin pedestals beneath the bacterial colonies (Supplementary Figure 2, arrow). By contrast, IEC infection with either the EPEC-Δ*tir* or the EPEC-TirY454F/Y474F mutants did not result in pedestal assembly (Supplementary Figure 2, arrowheads). NM IIA knockout IEC monolayers demonstrated a significantly increased attachment of EPEC-TirWT (Figure 7B and C), recapitulating our results obtained with the parental EPEC strain (Figure 4). However, IEC attachment of both pedestal formation-deficient Tir mutants was markedly lower when compared to the EPEC-TirWT (Figure 7B and C). More importantly, NM IIA knockout did not enhance the cell adhesion of the EPEC-Δ*tir* or the EPEC-TirY454F/Y474F mutants (Figure 7B and C). Similarly, the T3SS-deficient EPEC mutant showed very low IEC attachment, which was not affected by NM IIA knockout (Supplementary Figure 3). These data indicate that NM IIA limits EPEC attachment to IECs by attenuating bacterial-induced actin pedestal assembly.

### Loss of NM IIA inhibits EPEC-induced assembly of basal stress fibers in intestinal epithelial cells

How could NM IIA diminish the actin pedestal assembly of EPEC-infected IECs? Immunofluorescence labeling and confocal microscopy did not detect specific accumulation of NM IIA in the apical pedestals (Supplementary Figure 4). Paradoxically, NM IIC was found to be enriched in these structures (Supplementary Figure 4, arrow). The lack of specific pedestal accumulation of NM IIA indicates that this motor is unlikely to exert local control of the actin filament structure and dynamics in pedestals. We rationalized that NM IIA may regulate EPEC-induced global remodeling of the actin cytoskeleton, thereby limiting pedestal assembly. In addition to triggering the formation of apical actin pedestals, A/E bacteria are known to induce the assembly of basal stress fibers in host cells.^52^^,^^53^ Therefore, one could envision that these pathogen-induced apical and basal cytoskeletal structures antagonize each other's assembly by competing for monomeric actin, which is already in short supply even in normal cells.^54^ Immunofluorescence labeling demonstrated an accumulation of NM IIA by basal F-actin stress fibers in EPEC-infected HT-29 monolayers (Supplementary Figure 5, arrows). Significant colocalization between the NM IIA and F-actin signals at stress fibers was determined using the Pearson correlation and Mander's overlap coefficients, as described in the Materials and methods. Thus, it is possible that NM IIA-driven assembly and stabilization of basal F-actin structures decrease a supply of monomeric actin, hindering apical pedestal formation.

**Figure 8.**
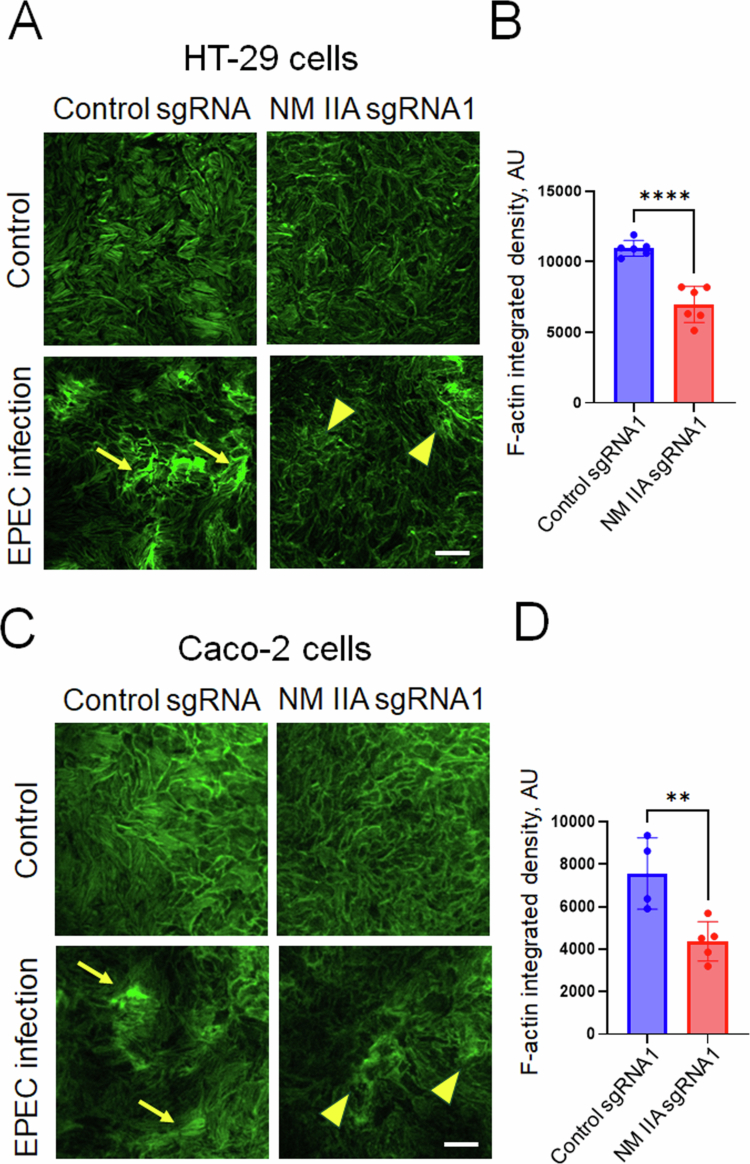
Loss of NM IIA attenuates basal stress fiber assembly in EPEC-infected intestinal epithelial cells. Control and NM IIA knockout HT-29cF8 (A and B) and Caco-2BBE (C and D) cells were infected with EPEC (MOI 5:1) for 3 h. The cells were fixed and fluorescently labeled for F-actin (green). Representative confocal images (A and C) and quantification of F-actin intensity at the cell base (B and D) are shown. Arrows point at basal stress fiber assembly in EPEC-infected control epithelial cell monolayers. Arrowheads indicate poor stress fiber assembly in the infected NM IIA knockout cells. Mean ± SEM, *n* = 4–6; **p < 0.01, ****p < 0.0001; scale bar = 20 μm.

We first tested the monomeric actin supply requirement by treating IECs with two small molecular compounds, known to regulate the equilibrium between monomeric and polymeric actin by different mechanisms. Latrunculin A decreases the supply of monomeric actin by sequestering such monomers and preventing their incorporation into the actin filaments.^55^^,^^56^ Cytochalasin D increases the supply of monomeric actin by promoting filament depolymerization at the pointed end while still allowing short filament assembly.^57^^,^^58^ While Latrunculin A treatment significantly reduced EPEC attachment, exposure to cytochalasin D increased bacterial adhesion to Caco-2 cell monolayers (Supplementary Figure 6A and B). Interestingly, fluorescence labeling of the actin cytoskeleton revealed a marked loss of actin filaments in latrunculin-treated IECs (Supplementary Figure 6C, arrows), while EPEC colonies were found to still be associated with a prominent actin cytoskeleton in cytochalasin-treated cells (Supplementary Figure 6C, arrowheads). This data suggests that bacterial interactions with IECs can be regulated by the availability of monomeric actin. Finally, we examined the formation of stress fibers in EPEC-exposed control and NM IIA-depleted IECs. Consistent with the data obtained in other experimental systems,^52^^,^^53^ EPEC infection of control HT-29 and Caco-2 cells induced a robust assembly of basal actin filament bundles (Figure 8A and C, arrows). By contrast, decreased basal F-actin bundle assembly was detected in EPEC-exposed NM IIA-knockout IECs (Figure 8A and C, arrowheads, B and D). Together, these data suggest that loss of NM IIA could redirect pathogen-induced actin cytoskeletal assembly from the basal stress fibers toward the apical pedestals, thereby promoting bacterial attachment to the IEC.

## Discussion

Attaching-effacing bacteria colonize mammalian host cells by triggering massive remodeling of the actin cytoskeleton, resulting in the assembly of actin pedestals beneath bacterial attachment sites.^2^^,^^10^^,^^15^ The formation of such apical pedestals involves a large number of different actin-binding and regulating proteins; however, the functional roles of only a small fraction of these proteins in regulating host‒pathogen interactions have been defined.^16^^,^^17^ Our study demonstrates for the first time that a key F-actin motor, NM IIA, acts as a negative regulator of A/E bacterial colonization of intestinal epithelial cells by interfering with actin pedestal assembly.

Our conclusion, highlighting NM IIA as a negative regulator of A/E pathogen interactions with intestinal epithelial cells, is based on the observation that either pharmacologic or genetic inhibition of this cytoskeletal motor increased EPEC attachment to model IEC monolayers *in vitro* (Figures 3 and 4). The physiological significance of this regulatory function of NM IIA is supported by our data showing increased *C. rodentium* colonization in mice with specific deletion of NM IIA in the intestinal epithelium (Figures 1 and 2). The expression of the NM IIA R702C mutant with diminished motor activity recapitulated the effects of NM IIA deletions in enhancing A/E bacterial interactions with the intestinal epithelium (Figure 6), further supporting the unique roles of the NM IIA motor in controlling host colonization by enteric A/E pathogens.

In contrast to the roles of NM IIA, we did not find NM IIC involvement in A/E bacterial infection, which is unexpected since this actin motor is ideally positioned to control enteric pathogen interactions with the intestinal epithelium. It selectively localizes at the apical pole of polarized IECs, being enriched in the perijunctional actomyosin belt.^22^^,^^59^ Furthermore, NM IIC is known to regulate microvilli dynamics at the apical pole of intestinal epithelial cells ^23^ and may therefore control microvilli effacement that is induced by attaching pathogens. Finally, our data suggests that NM IIC is enriched at EPEC pedestals (Supplementary Figure 4). Yet, either loss of NM IIC expression or its pharmacologic activation with 4-HAP had no effects on EPEC-interaction with IEC monolayers *in vitro* (Figures 3 and 5B, C), whereas total knockout of NM IIC in mice did not affect animal responses to *C. rodentium* colonization (Figure 5E–H).

Our study could help to resolve a long-standing uncertainty regarding the functional roles of NM II during A/E bacterial infections, which likely reflects the use of suboptimal experimental tools to interfere with myosin activity. Thus, previous pharmacological inhibition of MLC phosphorylation did not affect actin filament dynamics within the EPEC pedestals or pedestal motility on the epithelial surfaces.^60^ By contrast, studies utilizing butanedione monoxime (BDM) to block the ATPase activity of myosin II reported substantial elongation of actin pedestals in EPEC- and EHEC-infected epithelial cells.^24^^,^^60^ However, BDM is not a specific NM II inhibitor since it could also disrupt actin filament turnover.^61^ Therefore, the pedestal elongating activity of BDM could involve myosin-independent mechanisms. Subsequently, a report identified an EPEC effector, EspB, as an NM II-binding protein.^26^ Deletion of EspB was found to inhibit EPEC-induced microvilli effacement in Caco-2 cells and attenuate intestinal colonization by *C. rodentium* in mice.^26^ However, in addition to NM II, EspB also interacts with actin^62^ and unconventional myosins 1c, 5, 6, and 10.^26^ Therefore, the decreased host colonization by the EPEC mutant with deleted EspB cannot be attributed to the selective inhibition of NM II. Our study that combines pharmacologic and genetic inhibition of NM II motors demonstrates the increased intestinal epithelial colonization by A/E pathogens when NM IIA activity is compromised, both in cultured IEC *in vitro* and in mouse intestinal mucosa *in vivo*.

Importantly, our data suggests that NM IIA-dependent regulation of EPEC attachment to model IEC monolayers involves the assembly of bacterial pedestals. This conclusion is based on the observation that NM IIA deletion promoted the attachment of only EPEC strains with the functional Tir effector, which is capable of initiating actin pedestal assembly. By contrast, NM IIA inhibition did not affect the attachment of EPEC strains with either deletion of Tir or expressing the pedestal formation-deficient Tir mutant (Figure 7B and C).

How could NM IIA limit the assembly of apical actin pedestals? We did not find selective recruitment of NM IIA to pedestals in IECs (Supplementary Figure 4), indicating that this actin motor affects pedestal formation remotely. Such remote actions are likely to be linked to controlling the remodeling of other parts of the epithelial actin cytoskeleton triggered by attaching pathogens. Specifically, our data suggests that NM IIA could be essential for the pathogen-induced assembly of basal stress fibers. Stress fibers may aid bacterial colonization by increasing IEC attachment to the extracellular matrix and slowing down the extrusion of infected cells from epithelial monolayers. However, the simultaneous assembly of the apical pedestals and basal stress fibers could be antagonistic because of competition for monomeric actin, which is required to build both structures. Indeed, sequestration of monomeric actin with latrunculin A was inhibited, while an increase in the monomeric actin supply by cytochalasin D treatment accelerated EPEC attachment to IEC monolayers (Supplementary Figure 6A and B). The increased bacterial attachment in cytochalasin D-treated cells could be considered paradoxical since this compound is believed to prevent actin polymerization by capping the fast-growing barbed ends of actin filaments. However, our data suggest that, in contrast to latrunculin A treatment, cytochalasin D treatment does not disrupt epithelial actin filaments associated with attached EPEC colonies (Supplementary Figure 6C). Furthermore, a recent study that revisited the cellular activity of different cytochalasins revealed that these compounds do not completely block but rather remodel actin filament growth and could increase the association of short actin filaments with actin elongating Ena/VASP proteins.^57^ The combined effects of the enhanced actin monomer supply and increased recruitment of Ena/VASP proteins are likely to explain the increased EPEC attachment to cytochalasin D-treated IECs.

Robust inductions of basal actin stress fibers were evident in EPEC-infected control HT-29 cells (Figure 8), and these stress fibers were enriched in NM IIA (Supplementary Figure 5). Importantly, the assembly of EPEC-induced stress fibers was attenuated in NM IIA-deficient IEC monolayers (Figure 8). Taken together, these data suggest that NM IIA is essential for the assembly/stabilization of basal stress fibers induced by A/E pathogens. We hypothesize that attenuated stress fiber assembly in NM IIA-depleted IECs increases the availability of monomeric actin for the formation of apical actin pedestals, leading to increase A/E pathogen attachment. This idea is supported by a previous study showing that an EPEC effector protein, EspM, which induces stress fiber assembly, blocks actin pedestal formation in infected mammalian cells.^63^

While NM IIA directly modulates actin cytoskeletal remodeling to regulate A/E bacterial attachment to IEC monolayers *in vitro*, its effects on bacterial infection *in vivo* could be more complex. This suggestion is based on multiple homeostatic changes observed in NM II cKO mice. Specifically, unchallenged NM IIA cKO animals are characterized by increased permeability of the intestinal epithelial barrier and biochemical signs of mucosal inflammation (Figure 2B and C). Such abnormalities may result in significant alterations in both the intestinal microbiota and mucosa immune defense mechanisms of these animals. An altered gut microbiota and preactivated mucosal immunity could affect the colonization of NM II cKO mice with enteric A/E pathogens. These complex intestinal epithelial NM IIA-dependent mechanisms affecting host‒pathogen interactions *in vivo* could be an important focus of future studies.

In conclusion, the present study revealed a novel role of the NM IIA motor in inhibiting the interactions of A/E pathogens with the intestinal epithelium *in vitro* and *in vivo*. NM IIA serves as an essential regulator of bacteria-induced global remodeling of the actin cytoskeleton in IECs by attenuating the assembly of apical actin pedestals and accelerating the formation of basal stress fibers. This function requires the motor activity of the NM IIA and is not shared by its close paralog, NM IIC. Our findings open an opportunity for using pharmacological activation of NM II motors in order to decrease intestinal colonization by A/E pathogens.

## Disclosure of potential conflicts of interest

No potential conflicts of interest were disclosed.

## Acknowledgments

This work was supported by National Institutes of Health grant R01DK131550 to A.I.I. Confocal microscopy performed at the Cleveland Clinic Research Digital Imaging Microscopy Core utilized the Leica SP8 confocal microscope that was purchased with funding from the National Institutes of Health SIG grant 1S10OD019972-01.

We thank Dr. Michelle Dziejman (University of Rochester School of Medicine) for the insightful comments on the manuscript.

The results of this study were presented at the Pathobiology, Mechanisms of Disease 2024, Annual Meeting of the American Society for Investigative Pathology, and the summary of the results was published as an Abstract.^64^

## Supplementary Material

Supplementary material Suppl Fig5 Rev.tif

Supplementary material Suppl Fig6 Rev.tif

Supplementary material Suppl Fig2 Rev.tif

Supplementary material Suppl Fig4 Rev.tif

Supplementary material Suppl Fig1 Rev.tif

Supplementary material Suppl Fig3 Rev.tif

## Data Availability

Data available with the article and its supplementary materials. All relevant original data is publicly freely available in the Zenodo depository under DOI: 10.5281/zenodo.17387465.

## Abbreviations

A/E: attaching/effacing
cKO: conditional knockout
EPEC: Enteropathogenic *Escherichia coli*
IECs: intestinal epithelial cells
NM II: non-muscle myosin II
T3SS: type III secretion system
tKO: total knockout

## References

[1] Gruenheid S, Finlay BB. Microbial pathogenesis and cytoskeletal function. Nature. 2003;422:775–781. doi: 10.1038/nature01603. 12700772

[2] Stradal TEB, Schelhaas M. Actin dynamics in host-pathogen interaction. FEBS Lett. 2018;592:3658–3669. doi: 10.1002/1873-3468.13173. 29935019 PMC6282728

[3] Kaper JB, Nataro JP, Mobley HL. Pathogenic *Escherichia coli*. Nat Rev Microbiol. 2004;2:123–140. doi: 10.1038/nrmicro818. 15040260

[4] Kaur P, Dudeja PK. Pathophysiology of Enteropathogenic *Escherichia coli*-induced diarrhea. Newborn. 2023;2:102–113. doi: 10.5005/jp-journals-11002-0056. 37388762 PMC10308259

[5] Collins JW, Keeney KM, Crepin VF, Rathinam VAK, Fitzgerald KA, Finlay BB, Frankel G. *Citrobacter rodentium*: infection, inflammation and the microbiota. Nat Rev Microbiol. 2014;12:612–623. doi: 10.1038/nrmicro3315. 25088150

[6] Law RJ, Gur-Arie L, Rosenshine I, Finlay BB. In vitro and in vivo model systems for studying Enteropathogenic *Escherichia coli* infections. Cold Spring Harb Perspect Med. 2013;3:a009977–a009977. doi: 10.1101/cshperspect.a009977. 23457294 PMC3579205

[7] Huang CR, Kuo CJ, Huang CW, Chen Y, Liu B, Lee C, Chang W, Hsieh H. Host CDK-1 and formin mediate microvillar effacement induced by enterohemorrhagic *Escherichia coli*. Nat Commun. 2021;12:90. doi: 10.1038/s41467-020-20355-1. 33397943 PMC7782584

[8] Knutton S, Baldwin T, Williams PH, McNeish AS. Actin accumulation at sites of bacterial adhesion to tissue culture cells: basis of a new diagnostic test for Enteropathogenic and Enterohemorrhagic *Escherichia coli*. Infect Immun. 1989;57:1290–1298. doi: 10.1128/iai.57.4.1290-1298.1989. 2647635 PMC313264

[9] Shifrin DA, Jr., Crawley SW, Grega-Larson NE, Tyska MJ. Dynamics of brush border remodeling induced by Enteropathogenic *E. coli*. Gut Microbes. 2014;5:504–516. doi: 10.4161/gmic.32084. 25076126 PMC5642117

[10] Campellone KG. Cytoskeleton-modulating effectors of Enteropathogenic and Enterohaemorrhagic *Escherichia coli*: tir, EspFU and actin pedestal assembly. FEBS J. 2010;277:2390–2402. doi: 10.1111/j.1742-4658.2010.07653.x. 20477869

[11] Ruano-Gallego D, Sanchez-Garrido J, Kozik Z, Núñez-Berrueco E, Cepeda-Molero M, Mullineaux-Sanders C, Naemi Baghshomali Y, Slater SL, Wagner N, Glegola-Madejska I, et al. Type III secretion system effectors form robust and flexible intracellular virulence networks. Science. 2021;371. doi: 10.1126/science.abc9531. 33707240

[12] De Ryck J, Van Damme P, Goormachtig S. From prediction to function: current practices and challenges towards the functional characterization of type III effectors. Front Microbiol. 2023;14:1113442. doi: 10.3389/fmicb.2023.1113442. 36846751 PMC9945535

[13] Kenny B. Mechanism of action of EPEC type III effector molecules. Int J Med Microbiol. 2002;291:469–477. doi: 10.1078/1438-4221-00155. 11890546

[14] Pakbin B, Bruck WM, Rossen JWA. Virulence factors of enteric pathogenic *Escherichia coli*: a review. Int J Mol Sci. 2021;22(18):9922. doi: 10.3390/ijms22189922. 34576083 PMC8468683

[15] Caron E, Crepin VF, Simpson N, Knutton S, Garmendia J, Frankel G. Subversion of actin dynamics by EPEC and EHEC. Curr Opin Microbiol. 2006;9:40–45. doi: 10.1016/j.mib.2005.12.008. 16406772

[16] Miner MV, Rauch. Why put yourself on a pedestal? The pathogenic role of the A/E pedestal. Infect Immun. 2024;92(9):e0048923. doi: 10.1128/iai.00489-23. 38591884 PMC11384751

[17] Naydenov NG, Marino-Melendez A, Campellone KG, Marino‐Melendez A, Ivanov AI. Cytoskeletal mechanisms regulating attaching/effacing bacteria interactions with host cells: it takes a village to build the pedestal. Bioessays. 2024;46:e2400160. doi: 10.1002/bies.202400160. 39301984 PMC11502255

[18] Stradal TE, Costa SC. Type III secreted virulence factors manipulating signaling to actin dynamics. Curr Top Microbiol Immunol. 2017;399:175–199. 27744505 10.1007/82_2016_35

[19] Beach JR, 3rd., Hammer JA. Myosin II isoform co-assembly and differential regulation in mammalian systems. Exp Cell Res. 2015;334:2–9. doi: 10.1016/j.yexcr.2015.01.012. 25655283 PMC4433797

[20] Vicente-Manzanares M, Ma X, Adelstein RS, Horwitz AR. Non-muscle myosin II takes centre stage in cell adhesion and migration. Nat Rev Mol Cell Biol. 2009;10:778–790. doi: 10.1038/nrm2786. 19851336 PMC2834236

[21] Ivanov AI, Lechuga S, Marino-Melendez A, Marino‐Melendez A, Naydenov NG. Unique and redundant functions of cytoplasmic actins and nonmuscle myosin II isoforms at epithelial junctions. Ann N Y Acad Sci. 2022;1515:61–74. doi: 10.1111/nyas.14808. 35673768 PMC9489603

[22] Naydenov NG, Feygin A, Wang D, Kuemmerle JF, Harris G, Conti MA, Adelstein RS, Ivanov AI. Nonmuscle myosin IIA regulates intestinal epithelial barrier in vivo and plays a protective role during experimental colitis. Sci Rep. 2016;6:24161. doi: 10.1038/srep24161. 27063635 PMC4827066

[23] Chinowsky CR, Pinette JA, Meenderink LM, Lau KS, Tyska MJ, Bement W. Nonmuscle myosin-2 contractility-dependent actin turnover limits the length of epithelial microvilli. Mol Biol Cell. 2020;31:2803–2815. doi: 10.1091/mbc.E20-09-0582. 33026933 PMC7851865

[24] Law HT, Chua M, Moon KM, Foster LJ, Guttman JA. Mass spectrometry-based proteomics identification of Enteropathogenic *Escherichia coli* pedestal constituents. J Proteome Res. 2015;14:2520–2527. doi: 10.1021/acs.jproteome.5b00074. 25907766

[25] Sanger JM, Chang R, Ashton F, Kaper JB. Novel form of actin-based motility transports bacteria on the surfaces of infected cells. Cell Motil Cytoskeleton. 1996;34:279–287. doi: 10.1002/(SICI)1097-0169(1996)34:4<279::AID-CM3>3.0.CO;2-3. 8871815

[26] Iizumi Y, Sagara H, Kabe Y, Azuma M, Kume K, Ogawa M, Nagai T, Gillespie PG, Sasakawa C, Handa H. The Enteropathogenic *E. coli* effector EspB facilitates microvillus effacing and antiphagocytosis by inhibiting myosin function. Cell Host Microbe. 2007;2:383–392. doi: 10.1016/j.chom.2007.09.012. 18078690

[27] Singh AP, Sharma S, Pagarware K, Siraji RA, Ansari I, Mandal A, Walling P, Aijaz S. Enteropathogenic *E. coli* effectors EspF and Map independently disrupt tight junctions through distinct mechanisms involving transcriptional and post-transcriptional regulation. Sci Rep. 2018;8:3719. doi: 10.1038/s41598-018-22017-1. 29487356 PMC5829253

[28] Conlin VS, Wu X, Nguyen C, Dai C, Vallance BA, Buchan AMJ, Boyer L, Jacobson K. Vasoactive intestinal peptide ameliorates intestinal barrier disruption associated with *Citrobacter rodentium*-induced colitis. Am J Physiol Gastrointest Liver Physiol. 2009;297:G735–G750. doi: 10.1152/ajpgi.90551.2008. 19661153

[29] Manjarrez-Hernandez HA, Baldwin TJ, Aitken A, Williams P, Knutton S. Intestinal epithelial cell protein phosphorylation in Enteropathogenic *Escherichia coli* diarrhoea. Lancet. 1992;339:521–523. doi: 10.1016/0140-6736(92)90340-9. 1346880

[30] Philpott DJ, McKay DM, Mak W, Perdue MH, Sherman PM. Signal transduction pathways involved in enterohemorrhagic *Escherichia coli*-induced alterations in T84 epithelial permeability. Infect Immun. 1998;66:1680–1687. doi: 10.1128/IAI.66.4.1680-1687.1998. 9529098 PMC108105

[31] Yuhan R, Koutsouris A, Savkovic SD, Hecht G. Enteropathogenic *Escherichia coli*-induced myosin light chain phosphorylation alters intestinal epithelial permeability. Gastroenterology. 1997;113:1873–1882. doi: 10.1016/S0016-5085(97)70006-4. 9394726

[32] Hua Y, Wu J, Fu M, Liu J, Li X, Zhang B, Zhao W, Wan C. Enterohemorrhagic *Escherichia coli* effector protein EspF interacts with host protein ANXA6 and triggers myosin light chain kinase (MLCK)-dependent tight junction dysregulation. Front Cell Dev Biol. 2020;8:613061. doi: 10.3389/fcell.2020.613061. 33425920 PMC7785878

[33] Pal K, Nowak R, Billington N, Liu R, Ghosh A, Sellers JR, Fowler VM. Megakaryocyte migration defects due to nonmuscle myosin IIA mutations underlie thrombocytopenia in MYH9-related disease. Blood. 2020;135:1887–1898. doi: 10.1182/blood.2019003064. 32315395 PMC7243143

[34] Zhang Y, Conti MA, Malide D, Dong F, Wang A, Shmist YA, Liu C, Zerfas P, Daniels MP, Chan C, et al. Mouse models of MYH9-related disease: mutations in nonmuscle myosin II-A. Blood. 2012;119:238–250. doi: 10.1182/blood-2011-06-358853. 21908426 PMC3251230

[35] Campellone KG, Giese A, Tipper DJ, Leong JM. A tyrosine-phosphorylated 12-amino-acid sequence of Enteropathogenic *Escherichia coli* Tir binds the host adaptor protein Nck and is required for Nck localization to actin pedestals. Mol Microbiol. 2002;43:1227–1241. doi: 10.1046/j.1365-2958.2002.02817.x. 11918809

[36] Campellone KG, Leong JM. Nck-independent actin assembly is mediated by two phosphorylated tyrosines within Enteropathogenic *Escherichia coli* Tir. Mol Microbiol. 2005;56:416–432. doi: 10.1111/j.1365-2958.2005.04558.x. 15813734

[37] Velle KB, Campellone KG. Extracellular motility and cell-to-cell transmission of enterohemorrhagic *E. coli* is driven by EspFU-mediated actin assembly. PLoS Pathog. 2017;13:e1006501. doi: 10.1371/journal.ppat.1006501. 28771584 PMC5557606

[38] Velle KB, Campellone KG. Enteropathogenic *E. coli* relies on collaboration between the formin mDia1 and the Arp2/3 complex for actin pedestal biogenesis and maintenance. PLoS Pathog. 2018;14:e1007485. doi: 10.1371/journal.ppat.1007485. 30550556 PMC6310289

[39] Lechuga S, Cartagena-Rivera AX, Khan A, Cartagena‐Rivera AX, Crawford BI, Narayanan V, Conway DE, Lehtimäki J, Lappalainen P, Rieder F, et al. A myosin chaperone, UNC-45A, is a novel regulator of intestinal epithelial barrier integrity and repair. FASEB J. 2022;36:e22290. doi: 10.1096/fj.202200154R. 35344227 PMC9044500

[40] Mitchell DM, Ball JM. Characterization of a spontaneously polarizing HT-29 cell line, HT-29/cl.f8. In Vitro Cell Dev Biol Anim. 2004;40:297–302. doi: 10.1290/04100061.1. 15780006

[41] Lechuga S, Marino-Melendez A, Davis A, Zalavadia A, Khan A, Longworth MS, Ivanov AI. Coactosin-like protein 1 regulates integrity and repair of model intestinal epithelial barriers via actin binding dependent and independent mechanisms. Front Cell Dev Biol. 2024;12:1405454. doi: 10.3389/fcell.2024.1405454. 39040043 PMC11260685

[42] Bhinder G, Sham HP, Chan JM, et al. The *Citrobacter rodentium* mouse model: studying pathogen and host contributions to infectious colitis. J Vis Exp. 2013;72:e50222. 10.3791/50222 PMC3605715 23462619

[43] Lechuga S, Naydenov NG, Feygin A, Cruise M, Ervasti JM, Ivanov AI. Loss of beta-cytoplasmic actin in the intestinal epithelium increases gut barrier permeability in vivo and exaggerates the severity of experimental colitis. Front Cell Dev Biol. 2020;8:588836. doi: 10.3389/fcell.2020.588836. 33195251 PMC7644907

[44] Ivanov AI, Pero RS, Scheck AC, Romanovsky AA. Prostaglandin E(2)-synthesizing enzymes in fever: differential transcriptional regulation. Am J Physiol Regul Integr Comp Physiol. 2002;283:R1104–R1117. doi: 10.1152/ajpregu.00347.2002. 12376404

[45] Lechuga S, Baranwal S, Li C, Naydenov NG, Kuemmerle JF, Dugina V, Chaponnier C, Ivanov AI, Yamashita Y. Loss of gamma-cytoplasmic actin triggers myofibroblast transition of human epithelial cells. Mol Biol Cell. 2014;25:3133–3146. doi: 10.1091/mbc.e14-03-0815. 25143399 PMC4196865

[46] Wang S, Li S, Li Y, Jiang Q, Han J, Liu Y, Chen Y. Non-muscle myosin heavy chain 9 maintains intestinal homeostasis by preventing epithelium necroptosis and colitis adenoma formation. Stem Cell Reports. 2021;16:1290–1301. doi: 10.1016/j.stemcr.2021.03.027. 33891868 PMC8185465

[47] Straight AF, Cheung A, Limouze J, Chen I, Westwood NJ, Sellers JR, Mitchison TJ. Dissecting temporal and spatial control of cytokinesis with a myosin II inhibitor. Science. 2003;299:1743–1747. doi: 10.1126/science.1081412. 12637748

[48] Surcel A, Ng WP, West-Foyle H, Zhu Q, Ren Y, Avery LB, Krenc AK, Meyers DJ, Rock RS, Anders RA, et al. Pharmacological activation of myosin II paralogs to correct cell mechanics defects. Proc Natl Acad Sci U S A. 2015;112:1428–1433. doi: 10.1073/pnas.1412592112. 25605895 PMC4321244

[49] Hu A, Wang F, Sellers JR. Mutations in human nonmuscle myosin IIA found in patients with May-Hegglin anomaly and Fechtner syndrome result in impaired enzymatic function. J Biol Chem. 2002;277:46512–46517. doi: 10.1074/jbc.M208506200. 12237319

[50] Kovacs X, Conti Ma M, Ma X, Kovács M, Conti MA, Wang A, Zhang Y, Sellers JR, Adelstein RS. Nonmuscle myosin II exerts tension but does not translocate actin in vertebrate cytokinesis. Proc Natl Acad Sci U S A. 2012;109:4509–4514. doi: 10.1073/pnas.1116268109. 22393000 PMC3311360

[51] Gruenheid S, DeVinney R, Bladt F, Goosney D, Gelkop S, Gish GD, Pawson T, Finlay BB. Enteropathogenic *E. coli* Tir binds Nck to initiate actin pedestal formation in host cells. Nat Cell Biol. 2001;3(9):856–859. doi: 10.1038/ncb0901-856. 11533668

[52] Arbeloa A, Bulgin RR, MacKenzie G, Shaw RK, Pallen MJ, Crepin VF, Berger CN, Frankel G. Subversion of actin dynamics by EspM effectors of attaching and effacing bacterial pathogens. Cell Microbiol. 2008;10:1429–1441. doi: 10.1111/j.1462-5822.2008.01136.x. 18331467 PMC2610399

[53] Matsuzawa T, Kuwae A, Yoshida S, Sasakawa C, Abe A. Enteropathogenic *Escherichia coli* activates the RhoA signaling pathway via the stimulation of GEF-H1. EMBO J. 2004;23:3570–3582. doi: 10.1038/sj.emboj.7600359. 15318166 PMC516631

[54] Suarez C, Kovar DR. Internetwork competition for monomers governs actin cytoskeleton organization. Nat Rev Mol Cell Biol. 2016;17:799–810. doi: 10.1038/nrm.2016.106. 27625321 PMC5125073

[55] Coue M, Brenner SL, Spector I, Coué M, Korn ED. Inhibition of actin polymerization by latrunculin A. FEBS Lett. 1987;213:316–318. doi: 10.1016/0014-5793(87)81513-2. 3556584

[56] Spector I, Shochet NR, Kashman Y, Groweiss A. Latrunculins: novel marine toxins that disrupt microfilament organization in cultured cells. Science. 1983;219:493–495. doi: 10.1126/science.6681676. 6681676

[57] Lambert C, Karger M, Jiang X, Scholz J, Steffen A, Tang Y, Döring H, Geffers R, Stradal TE, Lappalainen P, et al. Differential interference with actin-binding protein function by acute cytochalasin B. Curr Biol. 2025;35:4684–4698. doi: 10.1016/j.cub.2025.08.023. 40914162

[58] Lin DC, Tobin KD, Grumet M, et al. Cytochalasins inhibit nuclei-induced actin polymerization by blocking filament elongation. J Cell Biol. 1980;84:455–460. doi: 10.1083/jcb.84.2.455. 6892916 PMC2110542

[59] Ivanov AI, Bachar M, Babbin BA, Adelstein RS, Nusrat A, Parkos CA, Cordes N. A unique role for nonmuscle myosin heavy chain IIA in regulation of epithelial apical junctions. PLoS One. 2007;2:e658. doi: 10.1371/journal.pone.0000658. 17668046 PMC1920554

[60] Shaner NC, Sanger JW, Sanger JM. Actin and alpha-actinin dynamics in the adhesion and motility of EPEC and EHEC on host cells. Cell Motil Cytoskeleton. 2005;60:104–120. doi: 10.1002/cm.20047. 15627283

[61] Forer A, Fabian L. Does 2, 3-butanedione monoxime inhibit nonmuscle myosin? Protoplasma. 2005;225:1–4. doi: 10.1007/s00709-004-0077-z. 15868207

[62] Hardwidge PR, Donohoe S, Aebersold R, Finlay BB. Proteomic analysis of the binding partners to Enteropathogenic *Escherichia coli* virulence proteins expressed in *Saccharomyces cerevisiae*. Proteomics. 2006;6:2174–2179. doi: 10.1002/pmic.200500523. 16552782

[63] Simovitch M, Sason H, Cohen S, Zahavi EE, Melamed-Book N, Weiss A, Aroeti B, Rosenshine I. EspM inhibits pedestal formation by enterohaemorrhagic *Escherichia coli* and Enteropathogenic *E. coli* and disrupts the architecture of a polarized epithelial monolayer. Cell Microbiol. 2010;12:489–505. doi: 10.1111/j.1462-5822.2009.01410.x. 19912240

[64] Naydenov NG, Lechuga S, Marino-Melendez A, Hammer JA, Campellone KG, Ivanov AI. Non-muscle myosin IIA regulates intestinal epithelial cell interactions with attaching-efffacing bacteria. Am J Pathol. 2024;194:S9–S9.

