# Supplementary figures and images for: Myosin IIA motor regulates attaching-effacing bacteria interactions with intestinal epithelium
Source: Gut Microbes. 2026 Feb 28;18(1):2638002. doi: 10.1080/19490976.2026.2638002 (PMC12959189; doi:10.1080/19490976.2026.2638002)

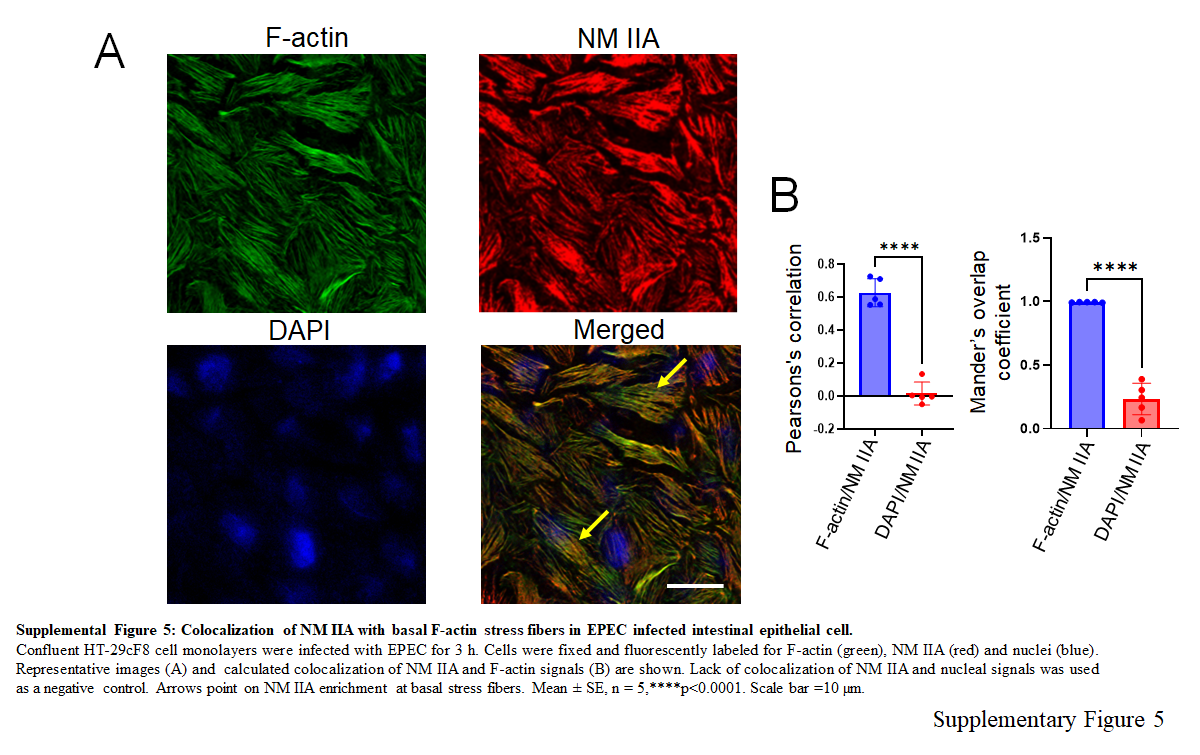

Supplement: Supplementary material — Suppl Fig5 Rev.tif [file KGMI_A_2638002_SM7617.tif]

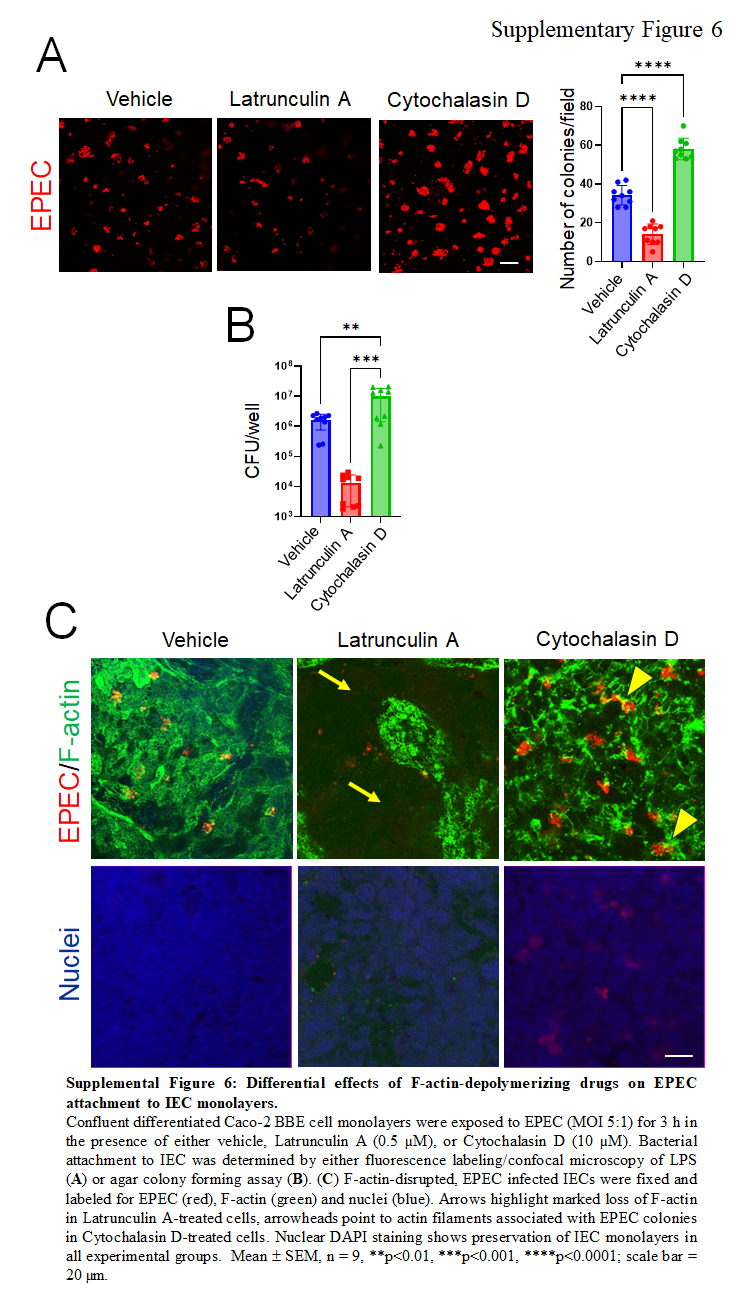

Supplement: Supplementary material — Suppl Fig6 Rev.tif [file KGMI_A_2638002_SM7612.tif]

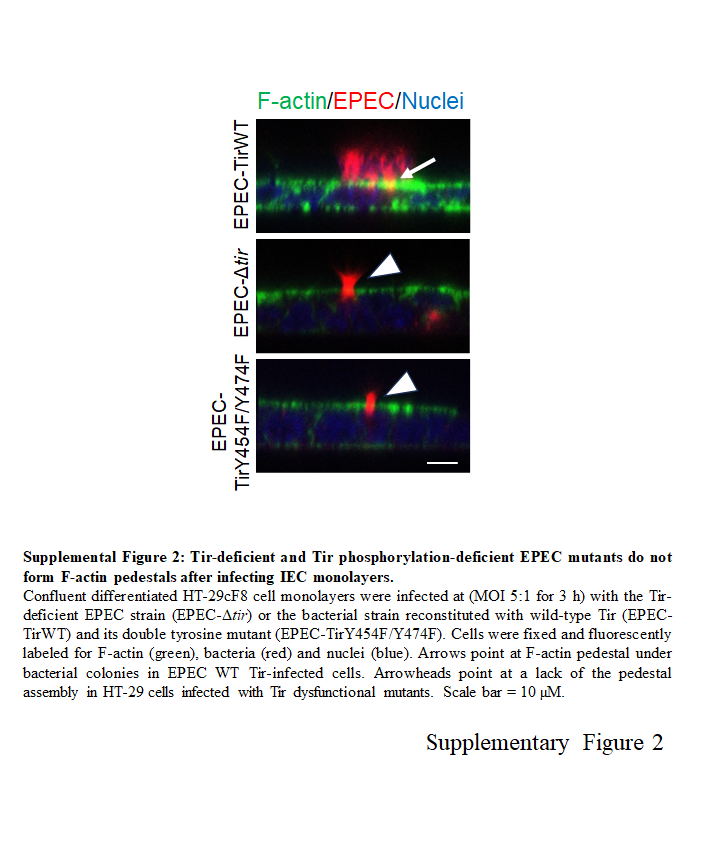

Supplement: Supplementary material — Suppl Fig2 Rev.tif [file KGMI_A_2638002_SM7613.tif]

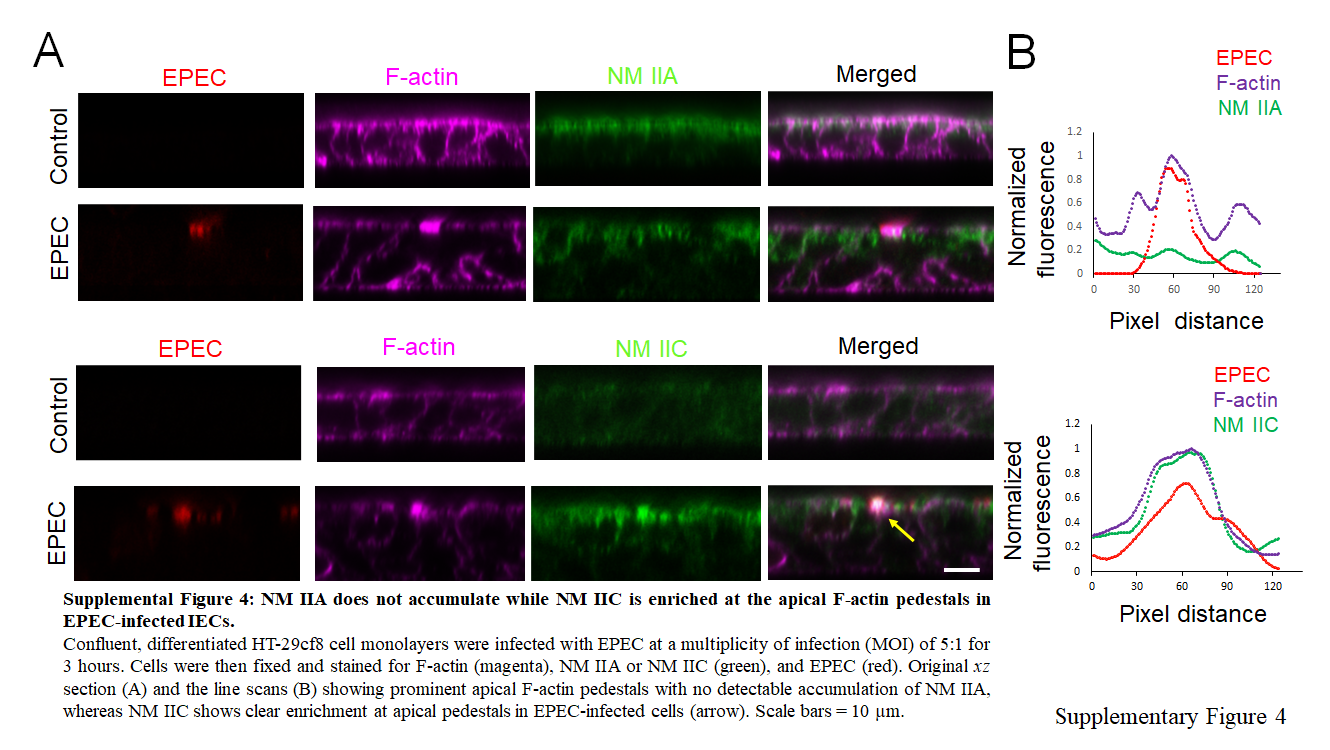

Supplement: Supplementary material — Suppl Fig4 Rev.tif [file KGMI_A_2638002_SM7614.tif]

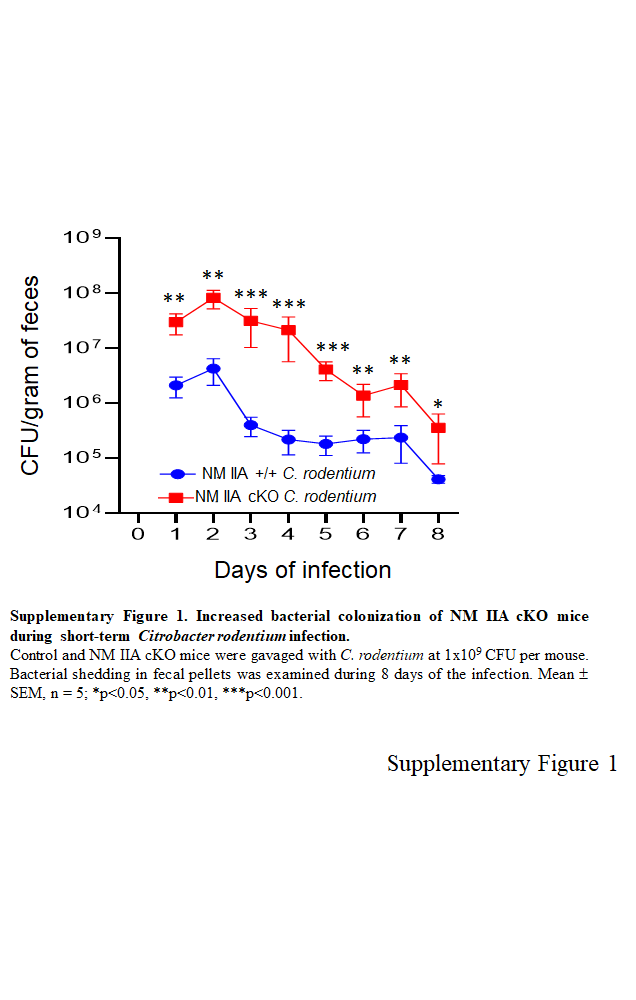

Supplement: Supplementary material — Suppl Fig1 Rev.tif [file KGMI_A_2638002_SM7615.tif]

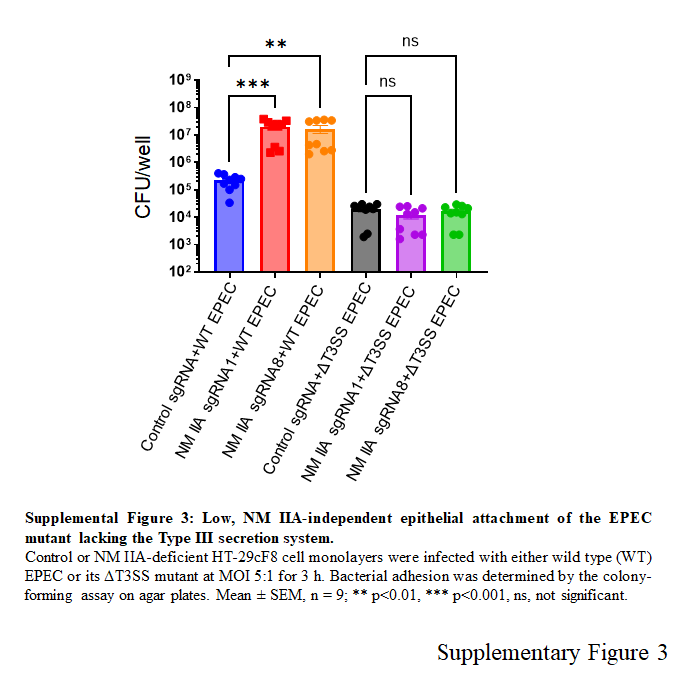

Supplement: Supplementary material — Suppl Fig3 Rev.tif [file KGMI_A_2638002_SM7616.tif]
